# Protection of CpG islands against *de novo* DNA methylation during oogenesis is associated with the recognition site of E2f1 and E2f2

**DOI:** 10.1186/1756-8935-7-26

**Published:** 2014-10-21

**Authors:** Heba Saadeh, Reiner Schulz

**Affiliations:** 1 Department of Medical & Molecular Genetics, King’s College London, 8th Floor Tower Wing, Guy’s Hospital, London SE1 9RT, UK; 2 Current address: Epigenetics Programme, The Babraham Institute, Babraham Research Campus, Cambridge CB22 3AT, UK

**Keywords:** Epigenetic reprogramming, CpG island, gene expression, DNA methylation, oogenesis, genomic imprinting, chromatin remodelling

## Abstract

**Background:**

Epigenetic reprogramming during early mammalian embryonic and germ cell development is a genome-wide process. CpG islands (CGIs), central to the regulation of mammalian gene expression, are exceptional in terms of whether, when and how they are affected by epigenetic reprogramming.

**Results:**

We investigated the DNA sequences of CGIs in the context of genome-wide data on DNA methylation and transcription during oogenesis and early embryogenesis to identify signals associated with methylation establishment and protection from *de novo* methylation in oocytes and associated with post-fertilisation methylation maintenance. We find no evidence for a characteristic DNA sequence motif in oocyte-methylated CGIs. Neither do we find evidence for a general role of regular CpG spacing in methylation establishment at CGIs in oocytes. In contrast, the resistance of most CGIs to *de novo* methylation during oogenesis is associated with the motif CGCGC, the recognition site of E2f1 and E2f2, transcription factors highly expressed specifically in oocytes. This association is independent of prominent known hypomethylation-associated factors: CGI promoter activity, H3K4me3, Cfp1 binding or R-loop formation potential.

**Conclusions:**

Our results support a DNA sequence-independent and transcription-driven model of *de novo* CGI methylation during oogenesis. In contrast, our results for CGIs that remain unmethylated are consistent with a model of protection from methylation involving sequence recognition by DNA-binding proteins, E2f1 and E2f2 being probable candidates.

## Background

Epigenetics encompass reversible biochemical modifications of DNA and chromatin that do not change the underlying DNA sequence but influence its interpretation by the cellular machinery, particularly with respect to gene expression. Epigenetic modifications are heritable across cell divisions so that cell type identity can be maintained [1]. On the other hand, alterations of epigenetic modifications are at the heart of cell lineage choices during differentiation and, thus, are critical in organism development [2]. DNA methylation (5-methyl-cytosine; 5mC) and post-translational modifications of histone tail residues are the epigenetic modifications most immediately linked to the control of mammalian gene expression, and their genome-wide profiles form cell type-specific combinatorial patterns [2].

Epigenetic reprogramming is required to generate the totipotent zygote, able to generate all embryonic and extra-embryonic cell types [2]. This is achieved by the erasure of epigenetic marks, DNA methylation in particular, on the genomes contributed by the germ cells and the subsequent establishment of new baseline marks [2,3]. Epigenetic reprogramming is also required during gametogenesis to uniformly set up the same, sex-specific epigenetic profile across all germ cell genomes, irrespective of their parental origin [2].

**Figure 1:**
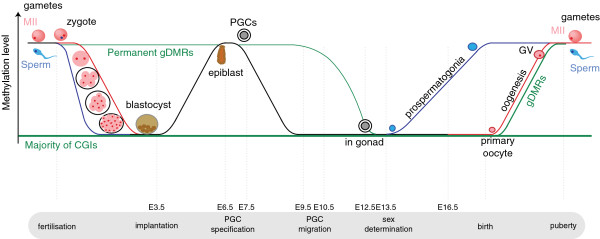
**DNA methylation reprogramming in pre-implantation mouse embryos (fertilisation to approximately E7.5) and the germ lines (approximately E7.5 to puberty).** The paternal genome undergoes rapid active genome-wide demethylation immediately after fertilisation, while slower passive demethylation (via DNA replication) affects the maternal genome. Methylation re-establishment occurs at around implantation and it affects both genomes. In the germ line, primordial germ cells lose their DNA methylation during early (black) and late (green) demethylation stages that affect different sequence categories, imprinted regions (permanent gDMRs) being among the late demethylated regions. While methylation is re-established in prenatal male germ cells, in oocytes this process does not complete until after puberty during their final growth stage due to being activated prior to ovulation. The paternal genome is shown in blue while the maternal genome is shown in red. The green lines refer to specific sets of CGIs: CGIs in imprinted regions that maintain allele-specific DNA methylation at least until implantation (permanent gDMRs), the majority of CGIs that maintain their methylation-free state, and CGIs that acquire methylation during oogenesis (gDMRs). The width of the line indicates the relative size of the respective CGI set. The black line represents both parental genomes. PGCs, primordial germ cells; GV, germinal vesicle oocyte; MII, metaphase II oocyte; E, embryonic day.

Immediately after fertilisation and when the parental genomes are still in their separate pronuclei, active genome-wide demethylation erases DNA methylation from the sperm-delivered paternal genome [4], though specific regions like, for example, paternally imprinted regions, maintain their methylation [2,5]. The oocyte-delivered maternal genome is initially protected from demethylation [5]. However, during subsequent cleavage divisions the maternal genome is demethylated passively, mainly due to the exclusion of Dnmt1 from the nucleus, with the exception of specific regions like, for example, maternally imprinted regions that remain methylated [2,5]. A minimum in terms of the overall genome-wide DNA methylation level is reached at the blastocyst stage, followed by a phase of genome-wide *de novo* DNA methylation that coincides with early embryonic differentiation and development, and completes at around the time of implantation (Figure 1).

In the germ line, DNA methylation and in particular parental genomic imprints are reset so that the genomes of mature gametes epigenetically reflect the sex of the individual. First, DNA methylation is actively removed from the genomes of primordial germ cells while they migrate to and colonise the genital ridge [6,7], a process that in the mouse completes at E13.5. The genomes of the developing germ cells are subsequently *de novo* re-methylated, a phase that in the male germ line is already complete prior to birth, while mouse oocytes do not undergo this process until after birth and only during their final growth phase, with *de novo* methylation completing between the germinal vesicle and meiosis II-arrested stages [8] (Figure 1).

The changes in DNA methylation levels during the periods of epigenetic reprogramming outlined above are overall genome-wide trends. Not all genomic regions follow these trends. An important exception are CpG islands (CGIs), CpG dinucleotide-dense regions between a few hundred and a few thousand nucleotides in length that are found at approximately 70% of mammalian gene promoters and play a central role in the regulation of gene expression [9]. The majority of the approximately 23,000 CGIs in the mouse genome identified by CAP-seq [10] are unmethylated, resisting *de novo* DNA methylation at all times, in contrast to most of the rest of the genome [11] (Figure 1). However, in the female germ line, approximately 1,600 CGIs (by extrapolation from the observed fraction of CGIs) acquire methylation during oogenesis [12,13]. Almost all of these oocyte-methylated CGIs remain unmethylated during spermatogenesis, that is, they are maternal germ line differentially methylated regions (maternal gDMRs). Almost all maternal gDMRs are transient in that maternal allele-specific methylation is lost during post-fertilisation reprogramming. However, there is yet another exceptional subset of 28 CGIs that are part of permanent maternal gDMRs, that is, those that are protected from post-fertilisation reprogramming and persist up to at least E8.5 [14]. Among these are almost all of the maternally methylated imprinting control regions (ICRs) that regulate imprinted, parental allele-specific gene expression [14].

DNA sequence features are known to play a role in epigenetic reprogramming [15,16]. We therefore hypothesised that the DNA sequences of the CGIs that acquire DNA methylation in the oocyte contain characteristic DNA sequence features. Using an ab initio DNA sequence motif discovery approach [17], we replicate and elaborate the previously experimentally determined highly specific association between the above permanent maternal gDMRs and the TGCCGC motif involved in their protection from post*-*fertilisation reprogramming [16]. The same, thus validated, approach fails to uncover a DNA sequence motif that is characteristic for oocyte-methylated CGIs in general. Those CGIs also do not exhibit a periodic pattern in the spacing of their CpGs, previously suggested to be involved in the targeting of *de novo* DNA methyltransferases [18]. In contrast, our ab initio approach identifies the CGCGC motif as a novel characteristic feature of those CGIs that are protected from *de novo* methylation during oogenesis. We show that high CpG density cannot explain this finding. Furthermore, the association of the CGCGC motif with the absence of DNA methylation at CGIs in the oocyte is independent of other factors, such as being an active promoter in the oocyte or having R-loop formation potential. We find that CGCGC is the recognition site of E2f1 and E2f2, transcription factors that, together with co-factors involved in chromatin remodelling, are highly expressed specifically in oocytes.

## Results and discussion

### An ab initio motif search identifies the Zfp57/Kap1 recognition site as a characteristic feature of permanent maternal gDMRs

**Figure 2:**
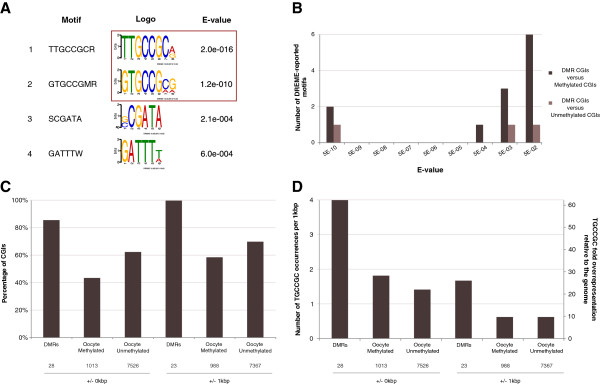
**TGCCGC motif analyses. (A)** DREME results for the DMR CpG islands (CGIs) (n = 28) versus oocyte-methylated CGIs (n = 1,013) comparison. Motif: a regular expression representation of the motif in IUPAC format. Logo: continuous motif representation (y-axis is information content in bits) on the forward strand. RC: reverse complement. E-value: statistical significance measure. **(B)** E-value distributions of DREME-identified motifs in DMR CGIs versus oocyte-methylated and oocyte-unmethylated CGIs. X-axis: E-value bins on a log_10_ scale, with text labels referring to the bin centres. Y-axis: number of motifs with an E-value in the respective bin. **(C)** Presence statistic for the TGCCGC motif per CGI category with and without +/- 1 kbp flanking sequences (x-axis). The y-axis represents the percentage of CGIs containing at least one occurrence of the motif. The numbers underneath each category are the total number of sequences in each CGI group. **(D)** TGCCGC density per CGI category with and without +/- 1 kbp flanking sequences. The primary y-axis represents density as occurrences per 1 kbp, while the secondary y-axis shows density relative to the background density in the whole mouse genome (NCBI build 37). DMRs, maternal permanent gDMR CGIs.

The DNA methylation on the methylated parental alleles of permanent gDMRs is maintained post-fertilisation by the Zfp57/Kap1 protein complex that recognises and binds to the methylated hexanucleotide TGCC^m^GC [16]. The motif was identified by overlaying the binding sites of Zfp57, Kap1 and Setdb1, identified by separate ChIP-seq experiments, and computing the consensus sequence over all sites occupied by all three proteins. As proof-of-principle for our computational approach, we wanted to determine if a purely DNA sequence-dependent (ab initio) motif discovery method [17] can reproduce the experimentally determined association between TGCCGC and imprinted regions, despite their limited number, and if so, what the statistical properties of this finding are. We thus compared the CGIs comprising permanent maternal gDMRs [see Additional file 1: Table S1], which for simplicity we refer to as DMR CGIs, with other classes of CGIs. Despite the limited number of sequences (n = 28), we found TGCCGC to be by far the most significantly (DREME [17]; E < 10^-9^) enriched motif in DMR CGIs, relative to CGIs that remain unmethylated in the oocyte (n = 7,526), as well as relative to oocyte-methylated CGIs that are not DMR CGIs (n = 1,013; Figure 2.A,B).

In terms of significance, six orders of magnitude separated TGCCGC from the next most significantly enriched distinct motif. We used this property of the E-value distribution in the case of TGCCGC as a benchmark in our subsequent ab initio motif searches. Specifically, we considered a motif to be characteristic of a sequence set if and only if it was reported as statistically significant (E <0.05) and constituted an extreme outlier in terms of significance compared to all other reported distinct motifs. Thus, a motif being characteristic implies high relative merit against the background of all reported motifs and, hence, increases the likelihood that the motif is a true positive finding. Reliance on only the significance metric reported by an ab initio method (for example, the DREME E-value) is prone to bias since the overall scale of the metric can vary widely depending, in a non-trivial manner, on variables like the input set sizes, sequence lengths and (di)nucleotide composition. The notion of a characteristic motif is generalisable to a characteristic (small) subset of reported motifs that all are extreme outliers in terms of significance. Such a subset comprising clearly distinct motifs could indicate the coordinate binding of multiple factors. However, in our experiments below, we did not encounter such subsets.

While being characteristic of DMR CGIs, TGCCGC is however still present in approximately 40% of the other oocyte-methylated CGIs so that the mere presence of TGCCGC is insufficient for protection from post-fertilisation demethylation (Figure 2.C), implying that other factors contribute to this mechanism. TGCCGC being the only characteristic motif renders unlikely that another DNA-binding protein (complex) with high specificity for a distinct recognition site is also involved. However, we observed that the enrichment of TGCCGC is not only due to a relatively large fraction of DMR CGIs in which it is present, but also due to a high density of occurrences (approximately 4 per 1 kbp; Figure 2.D) relative to other CGI groups. Consistent with the observations in [16], we find all DMR CGIs apart from *Slc38a4* harbour at least two instances of TGCCGC (there are two TGCCGC sites within 1 kbp of the *Slc38a4* gDMR). Overall, gDMR size is moderately correlated with the number of TGCCGC instances (r^2^ = 0.33; [see Additional file 1: Table S1]). This suggests that multi-occupancy by the Zfp57/Kap1 protein complex may be required for the permanent protection of a region.

### Oocyte-methylated CpG islands and promoters upstream do not harbour characteristic DNA sequence motifs

We hypothesised that the subset of CGIs that become methylated during oogenesis, unlike the vast majority of CGIs, may harbour a characteristic DNA sequence motif that would presumably have a role in targeting the *de novo* Dnmt3a/Dnmt3l DNA methylation complex. The same ab initio DNA sequence motif discovery approach as above did not identify any significantly enriched motifs and, hence, no characteristic motif in oocyte-methylated CGIs relative to oocyte-unmethylated CGIs. Given the association of CGI shores with tissue-specific methylation [19], we extended our search to CGIs including +/- 1 kbp of flanking sequence, which yielded sets of nominally significant motifs [see Additional file 2: Figure S1.A]. However, none of the motifs was characteristic as defined above. Moreover, the reported motifs were rich in TpG/CpA dinucleotides and devoid of CpGs [see Additional file 2: Figure S1.A], consistent with sequence evolution of oocyte-methylated CGIs driven by the high mutation rate of 5mC to T due to deamination (approximately tenfold greater rate than for any other substitution mutation) [20]. The results were essentially the same for CGIs including +/- 2 kbp of flanking sequence (data not shown), except that the number of reported motifs increased roughly twofold, and their significance values uniformly were an order of magnitude smaller. Hence, while the overall dinucleotide composition of the reported motifs likely reflects a genuine biological process, individually, each is unlikely to be a recognition sequence. The increases in the number of reported motifs and the simultaneously uniformly decreasing significance values upon including additional flanking sequence illustrate the above mentioned issue of bias in the significance metric when comparing two sets of sequences that increasingly and systematically differ in their dinucleotide composition.

In the absence of a characteristic motif within the oocyte-methylated CGIs themselves, we next examined the sequences of oocyte-active promoters whose transcripts extend across downstream oocyte-methylated CGIs. A detailed study of the *Gnas* locus demonstrated that transcription through the CGIs associated with the *Nespas* and *Gnas_exon1a* permanent maternal gDMRs is necessary for them to gain DNA methylation during oogenesis [21]. More recently, a significant positive association between CGI methylation in the oocyte and CGIs being intragenic relative to oocyte-expressed transcripts was observed in genome-wide data [13]. We therefore hypothesised that the oocyte-active promoters from which these transcripts originate may contain a characteristic sequence motif that is sufficient for the activity of these promoters, thus ensuring the methylation of the downstream CGI.

To avoid false positive results, we employed strict criteria to identify oocyte-active promoters from oocyte RNA-seq, BS-seq, RRBS-seq and H3K4me3 ChIP-seq data [12,13]. Briefly, transcripts were reconstructed using the Tuxedo protocol [22], and the region +/- 1 kbp around the TSS of a transcript was considered a promoter if it overlapped an unmethylated CGI and was enriched for H3K4me3. This set of promoter sequences was then split according to whether or not transcripts originating from the respective promoter contained an oocyte-methylated CGI. An ab initio motif search analogous to the above, comparing the two promoter sets (with a downstream oocyte-methylated CGI: n = 103; without: n = 2,017) did not identify any significantly enriched motifs. We then systematically expanded the promoter sequences to include +/- 2 kbp, +/- 4 kbp and +/- 5 kbp of sequence flanking the TSS, yielding three, six and eight nominally significant motifs, none of which was characteristic as defined above [see Additional file 2: Figure S1.B].

We note that the observed lack of characteristic sequence motifs in oocyte-methylated CGIs or promoters upstream does not rule out less parsimonious sequence-based models of DNA methylation establishment. For example, each of multiple distinct combinations of sequence motifs may be sufficient to induce DNA methylation. In theory, none of the individual combinations needs to form a characteristic set of motifs and, hence, all could evade detection by our ab initio approach.

### CpGs in oocyte-methylated CpG islands are not characteristically spaced

**Figure 3:**
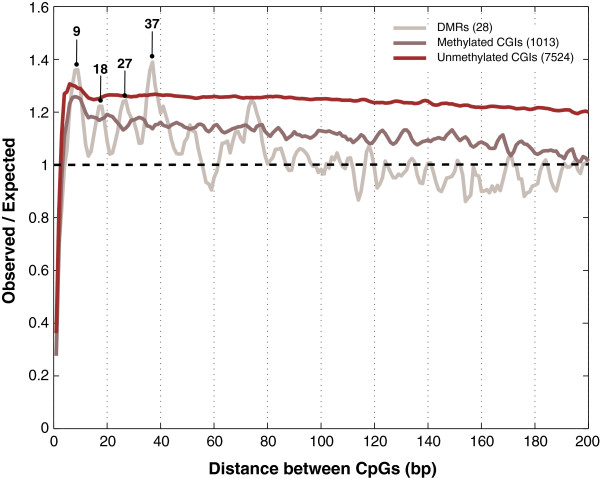
**Average observed/expected ratios for CpG pairs at distances up to 200 bp per CpG island (CGI) category.** Four enriched peaks are highlighted in DMR CGIs. The black dashed horizontal line (at value 1) indicates no difference between the observed and the expected values. The numbers between parentheses are the total number of sequences in each CGI group. DMRs, maternal permanent gDMR CGIs.

The Dnmt3a/Dnmt3l (Dnmt3a/l) tetramer protein complex that is responsible for *de novo* DNA methylation in oocytes has two active sites so that the complex can methylate two CpGs separated by 8 to 10 bp at the same time [18,23]. The authors of [18] also observed that the spacing of CpGs in twelve of the murine permanent maternal gDMRs on average exhibited a period of 8 to 10 bp, in contrast to ten CGIs on human Chr 21 and, therefore, suggested that this sequence feature may be a targeting signal for the recruitment of Dnmt3a/l. A subsequent investigation of this possibility by others provided only qualified support for this notion [24]. We asked the question whether the CpGs in CGIs that are *de novo* methylated by Dnmt3a/l in the oocyte are characteristically spaced.For the CGIs in each of our three categories (unmethylated CGIs, DMR CGIs, and oocyte-methylated CGIs that are not DMR CGIs), we computed the average observed over expected (obs/exp) ratio of the number of pairs of CpGs at a given distance, for distances up to 200 bp (see Methods for details). The average obs/exp ratios formed a periodic pattern for DMR CGIs but not for the other two CGI categories (Figure 3).

**Figure 4:**
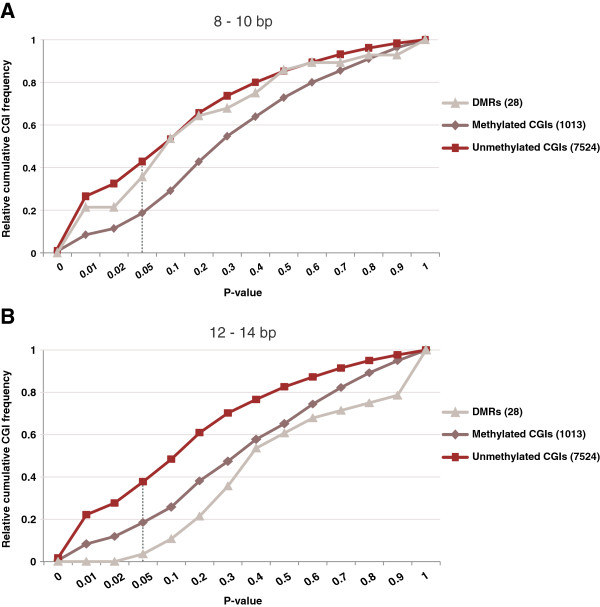
**Cumulative distributions of the empirical *P *value for the observed number of pairs of CpGs 8 to 10 bp (A) and 12 to 14 bp (B) apart.** The empirical *P* value (likelihood of observing as many or more CpG pairs at 8 to 10 bp/12 to 14 bp by chance) was determined for each CGI by comparison with 1,000 observations obtained by shuffling the CGI sequence while maintaining the dinucleotide frequencies. Here, we show the cumulative distributions of this *P* value among the CGIs in each of the three CGI categories (DMRs: DMR CGIs, other oocyte-methylated CGIs, and unmethylated CGIs). For a particular *P* value X, the cumulative distribution value Y equals the proportion of CGIs in the respective category with *P* < X. A vertical line marks X = 0.05, the *P* value threshold that we considered significant. At this threshold, 43%, 37% and 19% of unmethylated, DMR and other methylated CGIs, respectively, were significantly enriched with CpG pairs at 8 to 10 bp (A), versus 39%, 2% and 19% for CpG pairs at 12 to 14 bp. Overall, CpG pairs at 8 to 10 bp thus are enriched in DMR CGIs relative to CpG pairs at 12 to 14 bp, while no such distance-specific enrichment is observed for unmethylated or other methylated CGIs. This pattern is robust, that is, holds true for a wide range of *P* value thresholds.

Next, we determined for each CGI the significance of the observed number of pairs of CpGs at distances between 8 and 10 bp by comparison to the values obtained for 1,000 shuffled versions of the CGI sequence with dinucleotide frequencies identical to the original sequence [25]. We found that 43% of unmethylated CGIs are significantly (empirical *P* <0.05) enriched for pairs of CpGs at 8 to 10 bp, versus 37% of DMR CGIs and only 19% of the other oocyte-methylated CGIs (Figure 4.A). For pairs of CpGs at 12 to 14 bp, a distinct range of distances that does not include multiples of the 8 to 10 bp range, the results qualitatively differed only for DMR CGIs, only 2% of which were significantly enriched (Figure 4.B).

The smaller proportion (19%) of oocyte-methylated CGIs enriched for pairs of CpGs in relatively close proximity (8 to 10 bp and 12 to 14 bp) compared to unmethylated CGIs (39 to 43%) is consistent with the high mutation rate of 5mC [20] that over evolutionary time spans is expected to lead to lower CpG density. This is supported by the TG/CA-rich motifs identified above in oocyte-methylated relative to unmethylated CGIs including shores [see Additional file 2: Figure S1]. When we included CGI shores in the analysis of CpG pairs at 8 to 10 bp, the gap between oocyte-methylated and unmethylated CGIs became more pronounced [see Additional file 2: Figure S2], suggesting that the rate of CpG depletion is higher in the shores than in the cores of methylated CGIs. In vitro, Dnmt3a/l preferentially methylates CpG pairs at 8 to 10 bp [23]. Our results provide no evidence that CpG pairs at 8 to 10 bp are preferentially depleted in oocyte-methylated CGIs; that is, the in vitro preference of Dnmt3a/l is not obviously reflected in the sequence evolution of those CGIs.

The 28 DMR CGIs on average exhibit an approximately 9-bp period in CpG spacing and are enriched for CpG pairs at 8 to 10 bp relative to pairs at 12 to 14 bp (Figures 3 and 4). Individually however, they exhibit considerable variability with respect to the existence of periodic CpG spacing, as well as the lengths of the present periods, irrespective of the method used to assess periodicity [see Additional file 3: Supplementary Results and Methods; Additional file 1: Table S2; Additional file 2: Figures S3-S7]. This lack of consistency, even among CGIs belonging to the same permanent maternal gDMR, does not support a general involvement of periodic CpG spacing in targeting Dnmt3a/l to these regions.

Oocyte-methylated CGIs that are not DMR CGIs (the vast majority) lack periodic patterns in their average obs/exp ratios (Figures 3) and are equally depleted in CpG pairs at 8 to 10 bp and 12 to 14 bp (Figure 4). We conclude that regular CpG spacing, particularly with a period of 8 to 10 bp, is not associated with DNA methylation establishment by Dnmt3a/l at CGIs in the oocyte. Together with the lack of a characteristic DNA sequence motif in non-DMR oocyte-methylated CGIs, our results support a sequence-independent model of *de novo* DNA methylation during oogenesis.

**Figure 5:**
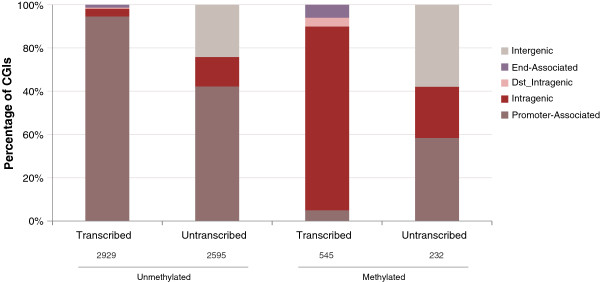
**Relationship between the DNA methylation state of CpG islands (CGIs) and their location relative to transcripts in the oocyte.** Almost all CGIs that are unambiguously associated with the promoter of an oocyte transcript are unmethylated. In contrast, a large majority of the CGIs that are unambiguously (distal) intragenic become methylated in the oocyte. CGIs that do not overlap oocyte transcripts were similarly classified according to their location relative to annotated genes (UCSC Known Genes). They illustrate that in the absence of actual transcription, there is no close relationship between CGI gene-relative position and oocyte methylation. The numbers underneath each category are the total number of CGIs in the respective class.

A sequence-independent model is compatible with the transcription elongation-driven model of DNA methylation establishment in the oocyte proposed in [21] and supported by the genome-wide results in [13]: of the CGIs associated with a transcript in the oocyte, 85% of the unmethylated CGIs were associated with a promoter, and 75% of the methylated CGIs (including DMR CGIs) were intragenic. We sought to replicate these findings, taking into account additional genome-wide data for oocytes [12]. We stratified CGIs according to their methylation state and position relative to transcripts and active promoters in the oocyte (Figure 5; see Methods for details). We excluded CGIs for which the classification was ambiguous, for example, CGIs associated with an active promoter as well as being intragenic relative to another transcript. We found almost all unmethylated CGIs (95%) associated with a transcript to be promoter-associated, and a large majority (85%) of the transcript-associated methylated CGIs were intragenic. However, though we observed an even greater degree of association between CGI methylation and intragenic location than previously reported, not all intragenic CGIs are methylated in the oocyte.

### The CGCGC DNA sequence motif is a characteristic feature of unmethylated CpG islands in the oocyte

**Figure 6:**
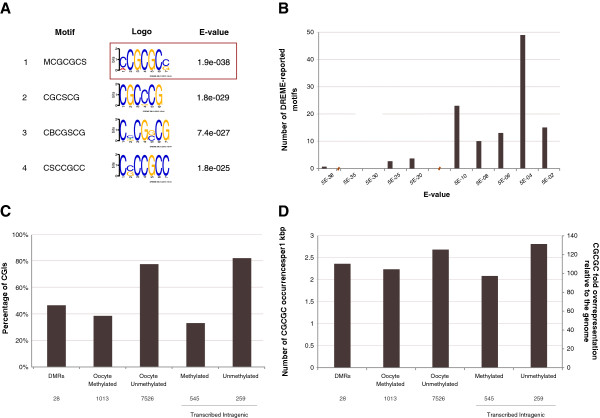
**CGCGC motif analyses. (A)** Motifs reported by DREME as the most significantly enriched in the set of intragenic unmethylated CpG islands (CGIs) (n = 259) compared to the set of intragenic methylated CGIs (n = 545). The CGCGC motif is the most significant motif with an extremely small E-value relative to the other reported motifs. **(B)** E-value distribution resulting from the *de novo* motif search comparing unmethylated intragenic with methylated intragenic CGIs that identified the CGCGC motif. The CGCGC motif is the short bar in the bottom-left corner, reported as nine orders of magnitude more significant than any of the other identified motifs. Similarly, TGCCGC was six to eight orders of magnitude more significant than any of the other reported motifs (Figure 2.B). **(C)** Presence statistic for the CGCGC motif per CGI category. **(D)** Density of the CGCGC motif per CGI category. DMRs, maternal permanent gDMR CGIs.

DNA methylation establishment during oogenesis results in the methylation of most gene body CpGs, while the CpGs comprising most CGIs remain unmethylated [12,13]. This includes a set of 259 CGIs that are intragenic relative to oocyte-expressed transcripts and, therefore, ought to be methylated given the model of transcription elongation-driven methylation [21]. Ab initio motif finding applied to the DNA sequences of these CGIs identified the motif MCGCGCS as significantly enriched (DREME E <10^-37^) in comparison to oocyte-methylated intragenic CGIs (Figure 6.A). Nine orders of magnitude separated the motif from the next most enriched motif so that like the Zfp57/Kap1 motif, it meets our criteria for a characteristic motif (Figure 6.B). The result for repeat-masked sequences was almost the same (CGCGCS; [see Additional file 2: Figure S8.A]). For simplicity, in the text below, we refer to the motif by its core sequence: CGCGC. The motif is present in 82% of unmethylated intragenic CGIs versus in 33% of oocyte-methylated intragenic CGIs and, more generally, in 77% of unmethylated CGIs versus 38% of oocyte-methylated CGIs (Figure 6.C). The density of occurrences also is higher in unmethylated CGIs (Figure 6.D).

The pattern of CGCGC motif density values across the different CGI categories is very similar to the pattern for the CpG dinucleotide [see Additional file 2: Figure S8.B], which raised the question of whether the motif occurrences are a simple consequence of the greater CpG density of unmethylated CGIs. To test this hypothesis, we shuffled the sequences of the unmethylated CGIs, while maintaining dinucleotide frequencies as above [25], and subsequently determined the occurrences of the motif. We observed a approximately 45% reduction in the number of motif occurrences, almost doubling the number of unmethylated CGIs without a motif occurrence and reducing the overall density of occurrences by 38% [see Additional file 2: Figure S8.C-E]. This rules out the globally high CpG density of unmethylated CGIs as the cause of the motif occurrences. However, CpGs are typically not uniformly distributed within a CGI, so the local CpG density varies within a CGI. Thus, locally high CpG density may explain the motif occurrences, or at least a large fraction of them. To test this possibility, we determined the distribution of the number of motif occurrences as a function of local CpG content and compared the distribution obtained for the unmethylated CGIs with the distribution for their shuffled counterparts [see Additional file 2: Figure S9]. Only approximately 5% of the motif occurrences in unmethylated CGIs can be explained by high local CpG density. In conclusion, repetitive sequence elements and high CpG density are unlikely explanations for the enrichment of the CGCGC motif in unmethylated CGIs.

### The significant association of the CGCGC motif with unmethylated CpG islands in the oocyte is independent of other, known hypomethylation-associated factors

Previous work by others has associated the typical lack of DNA methylation at CGIs with several factors. Promoter activity of a CGI is generally thought to be incompatible with CGI methylation [9,26]. R-loop formation potential of a CGI has been reported as a distinguishing feature of unmethylated CGIs in human embryonic stem cells (ESCs) [27]. In mouse ESCs, fibroblasts and brain, the binding of Cfp1 to CGIs is associated with hypomethylation via recruitment of the Set1 histone methyltransferase complex that deposits H3K4me3 [28,29]. H3K4me3 in turn inhibits the Dnmt3a/l complex and, thus, is directly associated with hypomethylation [30,31]. Here, we investigated these factors together with the CGCGC motif as a novel fifth factor to estimate the relative strengths of their association with hypo-methylation of CGIs in the oocyte, and their inter-dependencies.

We took a logistic linear regression approach, modelling the binary methylation state (either methylated or unmethylated) of 8,567 CGIs in the oocyte as linear combinations of subsets of binary factors and interaction terms. We refer to the five factors as PA, Rloop, Cfp1, H3K4me3 and Motif, and they are defined as follows: the CGI is/is not an active promoter in the oocyte (PA), the CGI has/does not have R-loop formation potential (Rloop), the CGI is/is not bound by Cfp1 in mouse whole brain tissue (Cfp1), the CGI is/is not enriched for H3K4me3 in the oocyte (H3K4me3), and the CGI does/does not contain the CGCGC motif (Motif). We note that R-loop formation potential, as opposed to actual R-loop formation, is a DNA sequence- and oocyte transcriptome-derived feature, termed G-skew in [27], that is, more G than C residues in the transcribed strand of a CGI. Thus, apart from Cfp1, all factors incorporate cell type-independent sequence and/or oocyte-specific experimental data. The set of factors was non-redundant since pairwise correlation between factors did not exceed 0.62 and typically was <0.3 [see Additional file 1: Table S3]. The values for all factors for all CGIs are part of Additional file 4 and Additional file 5 (‘Un-methylated associated Factor’ spreadsheet).

First, we determined which of the five factors in isolation have significant predictive value (reduction in model deviance) in terms of predicting the methylation state of a CGI in the oocyte. All factors had significant predictive value. In terms of effect size (reduction in deviance), the CGCGC motif was second after H3K4me3, and R-loop formation potential was a distant last [see Additional file 1: Table S4]. This is in agreement with the observed levels of correlation between the methylation state and each of the factors [see Additional file 1: Table S3].

**Figure 7:**
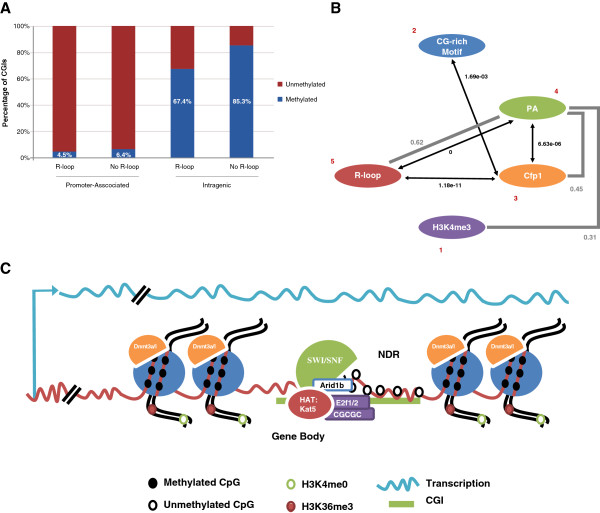
**Analysis of hypomethylation-associated factors. (A)** R-loop formation potential in distinct CpG island (CGI) groups. The percentages of methylated (blue) versus unmethylated (red) CGIs within each category are shown. The effect of R-loop formation potential on DNA methylation is small once CGIs have been stratified into promoter associated (PA) versus intragenic groups. **(B)** Pairwise significant interaction/correlation network of hypomethylation-associated factors. The numbers from 1 to 5 (red) indicate the rank of the factors based on the reduction in deviance compared to the null model which represents their power in predicting CGI methylation state (1 = most predictive value; Table S4). Significant interactions between factors are represented by black lines labelled with the Bonferroni multiple testing corrected chi-square *P* value (see Table S5 for a complete list of pairwise interaction statistics). Factors that are correlated with an absolute Pearson correlation coefficient (CC) >0.29 (the maximum correlation observed between any of the factors and the methylation state) are connected by grey lines labelled with the CC (see Table S3 for a complete list of pairwise CCs). **(C)** The proposed model of how a methylation-free state is established and maintained at oocyte-unmethylated CGIs. We propose that chromatin remodelling (Swi/Snf via Arid1b) and histone acetyltransferase (Kat5) complexes protect the CGI sequence from *de novo* methylation by keeping the CGI nucleosome-free and, therefore, free of the preferred substrate of Dnmt3a/l. They, in turn, are recruited to the CGI by E2f1 and/or E2f2 bound to the CGCGC recognition site in the CGI sequence.

In [27], 65% of human promoter CGIs versus 16.3% of intragenic CGIs (promoter/intragenic labels derived from gene annotation) and 67.4% of unmethylated (in human ESCs) CGIs versus 9.4% of methylated CGIs were observed to have R-loop formation potential. The authors concluded that ‘unmethylated CGI promoters are highly associated with strong GC skew and, therefore, with significant R loop formation potential’. Our finding that R-loop formation potential is a relatively poor predictor of the CGI methylation state therefore was surprising. In mouse oocytes, we find that while 67% of the CGIs at active promoters have R-loop potential, 54% of the intragenic CGIs do also. Similarly, while 67% of CGIs that remain unmethylated in the oocyte have R-loop potential, so do 51% of oocyte-methylated CGIs. The former has a potential explanation in that a substantial fraction of CGIs that are intragenic in the oocyte may coincide with annotated promoters, but we found that to be true for only approximately 11% of the intragenic CGIs. The latter is difficult to reconcile with [27] because the results do not rely on annotation database content. Since the promoter-associated versus intragenic labels of CGIs are highly correlated with their methylation state in the oocyte (Figure 5), we stratified CGIs according to the label and separately determined the effect of having versus not having R-loop formation potential on the fraction of methylated CGIs (Figure 7.A). While R-loop formation potential decreases the fraction of methylated CGIs within both the promoter-associated and the intragenic CGI categories, the reductions and associated odds-ratios (OR) are relatively small: 2% (OR: 1.5) for promoter-associated, and 18% (OR: 2.8) for intragenic CGIs. For comparison, the CGCGC motif reduces the fraction of methylated CGIs by 9% (OR: 4.0) and 37% (OR: 8.8), respectively [Additional file 2: Figure S10.A]. We conclude that R-loop formation potential on its own confers relatively little protection against DNA methylation in the oocyte.

We next tested whether the addition of the Motif factor to a model comprising one of the other factors significantly improved model fit and, hence, whether the CGCGC motif conveys significant additional, independent power to predict the methylation state. We found this to be true for all pairwise combinations of the Motif factor with one of the other factors [see Additional file 1: Table S4; Additional file 2: Figure S10.B]. This suggests that the presence of the CGCGC motif in a CGI independently confers additional protection from DNA methylation in the oocyte, in particular independent of promoter activity.

Finally, we tested each pair of factors for significant interaction, that is, a significant increase in the predictive value of the model upon the addition of an interaction term to the model composed of the two factors (Figure 7.B). The most significant interaction with by far the largest effect size occurs between promoter-association and R-loop formation potential [see Additional file 1: Table S5], consistent with promoter activity being required to realise actual R-loop formation at CGIs that have the potential to do so, and consequently, significant extra protection from DNA methylation above and beyond the effect of R-loop potential or promoter-association alone. The effect sizes of all other significant pair-wise interactions between factors were relatively small. That includes the only significant interaction between the CGCGC motif and another factor, namely, Cfp1 binding.

### The CGCGC motif matches the recognition site of E2f1 and E2f2, DNA-binding proteins involved in chromatin remodelling

We searched the Jaspar and Uniprobe motif databases for matches of the CGCGC motif to previously reported recognition sites of DNA-binding proteins. The yeast proteins RSC3 and RSC30, and the mammalian proteins E2f1, E2f2, E2f3 and Zfp161, have significantly (FDR <20%) matching database entries [see Additional file 2: Figure S11]. The match to E2f1 is supported further by an E2F1 ChIP-seq experiment in human MCF7 cells that identified CGCGC as the consensus binding sequence [32]. Transcriptionally, *E2f1* and *E2f2* are highly expressed specifically in oocytes (>15x of the median expression level across tissues), in contrast to *E2f3* and *Zfp161* (*aka Zbtb14*) ([see Additional file 2: Figure S12], [33] and also [34]).

We re-analysed the E2F1 ChIP-seq data from [32], identifying 30,467 sites of significant E2F1 enrichment (over input) and [GG]CGCGC as the most significantly enriched motif see Additional file 6: Mini-website with GEM results]. Almost 2/3 of the E2F1 binding sites overlap a CGI from [10]. Using the transcripts annotated by UCSC Known Genes (see Methods for details), we determined that E2F1 is >55x more likely (lower bound of odds ratio (OR) 95% confidence interval) to bind a CGI promoter than a non-CGI promoter (Fisher’s exact test; *P* <10^-15^); similarly for the comprehensive set of Gencode v19 transcripts (OR >72; *P* <10^-15^). However, only between 8,517 (UCSC) and 8,814 (Gencode) promoters are expressed in MCF7 cells (FANTOM5 CAGE: >1 tags per million mapped tags (TPM)). Still, even among only the expressed promoters, E2F1 has a strong preference for CGI promoters (OR >9.4 (UCSC), >14.2 (Gencode); *P* <10^-15^).

**Figure 8:**
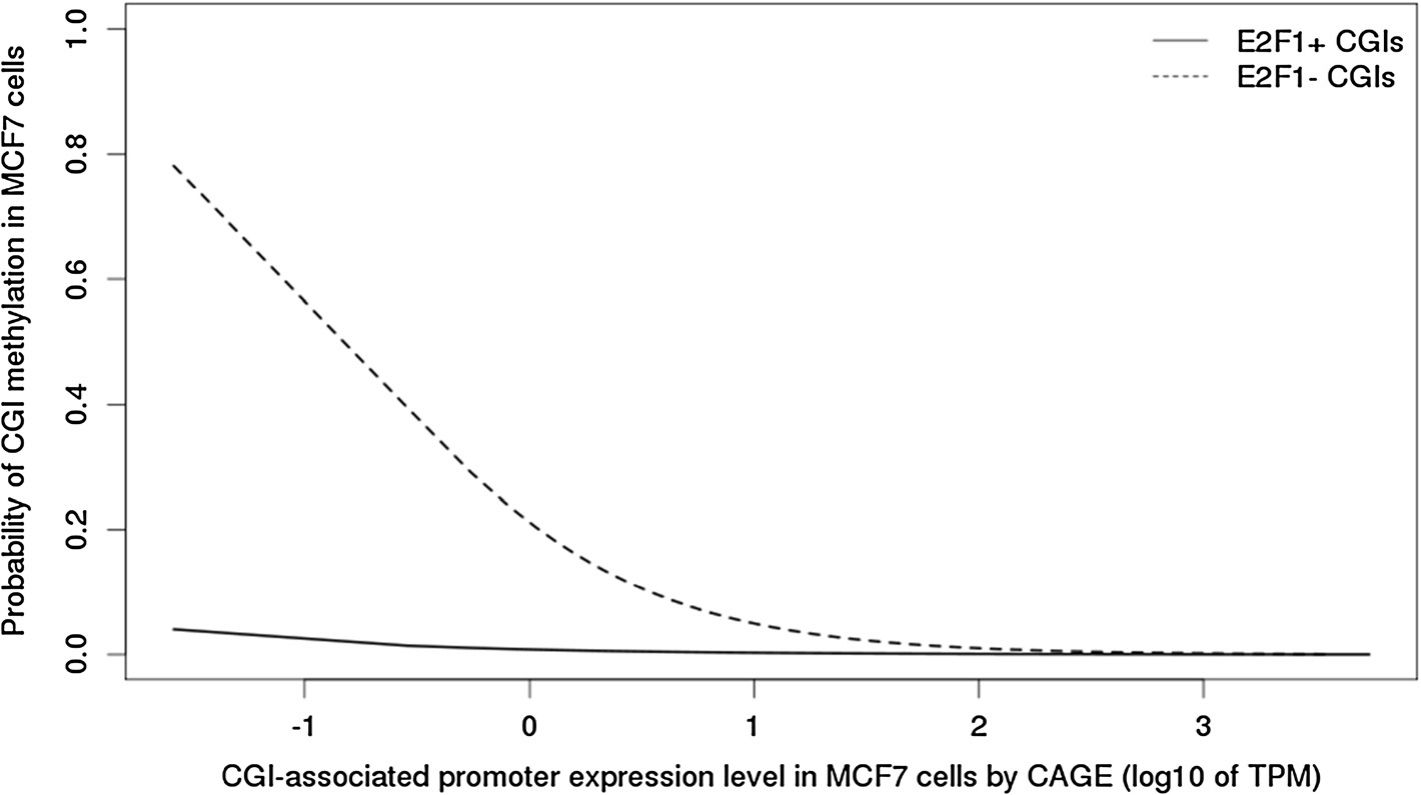
**E2F1 binding of CpG islands (CGIs) in MCF7 cells is associated with lack of CGI methylation, independent of CGI promoter activity.** We fitted a logistic regression model to MCF7-specific data on the methylation state (M in {0, 1}; derived from ENCODE RRBS-seq; see main text), promoter activity (P = log_10_ of maximum TPM observed by FANTOM5 CAGE) and E2F1 binding (E2F1 in {0, 1}; derived from [32]; see Methods) for 12,358 unmethylated (M = 0) and 4,427 methylated (M = 1) CGIs. All coefficients of the fitted model, M = logit(-1.31 -1.63*P -3.49*E2F1 + 0.60*P*E2F1), were of significant magnitude (*P* <10^-7^), that is, promoter activity and E2F1 binding both were independently and significantly negatively associated with CGI methylation, the degree of association being approximately 10x greater for E2F1. All terms of the model, including the interaction term, significantly improved model fit (chi-squared *P* <10^-6^). The plot shows the relationship between the level of CGI promoter activity (x-axis) and the probability of the CGI being methylated as predicted by the fitted model (y-axis), separately for CGIs bound by E2F1 (E2F1+) versus for CGIs not bound by E2F1 (E2F1-). The probability is essentially zero for CGIs bound by E2F1, irrespective of the level of their promoter activity, while in the absence of E2F1, the methylation state depends on the level of promoter activity, with only active CGIs (x >0: TPM >1) dropping below the 20% methylation threshold (y <0.2) typically considered as unmethylated.

Given the CGI preference of E2F1, we checked that CGIs containing the CGCGC motif are indeed more likely bound by E2F1 (OR >4.5; *P* <10^-15^). We then used ENCODE RRBS-seq data for MCF7 cells to determine the methylation state of CGIs: of the CGIs with sufficient read coverage (see Methods for details), 12,358 were unmethylated (<20% median per-CpG methylation), 4,427 were methylated (>80%), and 1,468 were hemimethylated. Relating the CGI methylation state to E2F1 binding, we found E2F1 > 133x more likely to bind to unmethylated versus methylated CGIs (*P* <10^-15^). Using logistic regression, we then modelled the methylation state of a CGI in terms of its promoter activity and E2F1 binding. Promoter activity and E2F1 binding were both significantly negatively associated with CGI methylation (*P* <10^-15^), the association being approximately 10x stronger for E2F1 binding (OR = 0.03 versus OR = 0.2 for promoter activity). Plotting of CGI promoter activity versus the model prediction of CGI methylation probability, stratified by E2F1 binding, revealed that E2F1 binding in MCF7 cells is associated with a lack of CGI methylation irrespective of the degree of CGI promoter activity (Figure 8), consistent with our CGCGC motif-based analysis for oocytes.

E2f1-3 are considered ‘activators’ since they induce H3 and H4 acetylation at target promoters [35]. DNA binding of E2f1 is required in particular for H4 acetylation, and E2f1 directly interacts with the Kat5 (aka Tip60) [35] histone acetyltransferase (HAT) complex whose preferred targets include K5, K8, K12 and K16 of H4 [36]. This was observed in non-dividing cells, that is, the results are relevant for oocytes where *Kat5* also is highly expressed [34].

The E2f family are also known to interact with the Swi/Snf chromatin remodelling complex via Arid1a and Arid1b [37]. Genes encoding Swi/Snf components (*Smarca2, Smarcb1, Smarcc2, Smarce1, Actl6a*), and *Arid1a* as well as *Arid1b* are highly expressed in mouse oocytes [33,34,38,39]. Like *E2f1* and *E2f2*, *Arid1b* is highly expressed specifically in oocytes [see Additional file 2: Figure S12] [33]. E2f1 and Kat5 specifically interact with Arid1b, and in proliferating cells, Arid1b is required for the binding of Swi/Snf to the promoters of cell-cycle-specific genes [37]. The Swi/Snf complex can move nucleosomes along DNA, and its recruitment to nucleosomes is enhanced by histone acetylation [40].

Nucleosome-bound DNA is the preferred substrate of the Dnmt3a/l complex, consistent with features of its structure and the generation of strand-asymmetric pairs of 5mC by its two active sites that are approximately 9 bp apart [18,23]. In addition, Dnmt3a has particularly high affinity for H3K36me3-marked nucleosomes [41,42]. H3K36me3 follows transcriptional elongation [43], and while overall being associated with deacetylation [44], there is complex interplay with H4K16 acetylation [45] along transcribed genes.

In this wider context, our findings support a model (Figure 7.C) of E2f1 and/or E2f2 contributing to sequence-specific protection of CGIs from *de novo* DNA methylation in the oocyte via the recruitment of Kat5 and Swi/Snf, the latter removing nucleosomes from the CGI and thus inhibiting Dnmt3a/l activity on the CGI sequence, even if transcription proceeds through the CGI, which would normally lead to DNA methylation. Our analysis results for human MCF7 cells indicate that E2f1 may play a role in the regulation of DNA methylation at CGIs in somatic cell types too.

Definitive proof of such a role for E2f1/2 will require the genome-wide assessment of DNA methylation in (oocyte-conditional) *E2f1/2* knock-outs, direct observation of E2f1/2 binding in oocytes, and/or DNA methylation studies of specific loci with engineered deletions or insertions of E2f1/2 recognition sites. Homozygous triple knock-out mice for *E2f1*, *E2f2* and a non-canonical isoform of *E2f3* survive to adulthood, the only described phenotype being a lower body weight [46]. While these are unconditional knock-outs, they may provide an opportunity to study the effects of E2f1 and E2f2 deficiency on DNA methylation in oocytes and during embryogenesis. Transcription factor ChIP-seq in oocytes remains a technical challenge, but given the known E2f1 recognition motif, other, less demanding methods may prove effective at identifying *bona fide* E2f1 binding sites in oocytes [47].

## Conclusions

Our results support a sequence-independent and transcription elongation-driven model of *de novo* CGI methylation during oogenesis. However, the vast majority of CGIs resist *de novo* DNA methylation during oogenesis, and we show that this resistance is associated with the CGCGC DNA sequence motif. The motif is the recognition site consensus for two DNA-binding proteins, E2f1 and E2f2, involved in chromatin remodelling via Arid1b and the Swi/Snf complex. *E2f1*, *E2f2* and *Arid1b* are highly expressed specifically in oocytes. On the basis of our results in this context, we propose that sequence-specific E2f1 and/or E2f2 binding to CGIs in the oocyte confers protection against *de novo* DNA methylation via nucleosome depletion by recruited Swi/Snf.

## Methods

### CpG island, gDMR and oocyte promoter coordinates

CGI coordinates, relative to mouse genome NCBI build 37, were taken from [13]. Permanent maternal gDMR coordinates were taken from [14]. Promoter regions of oocyte-expressed transcripts were defined as +/- 1 kbp around the transcription start site (the start of the first exon) of a Cufflinks-reconstructed transcript for which Cufflinks was able to determine the strand of origin and hence, the direction of transcription. Promoter regions also had to overlap a CGI and a region of H3K4me3-enrichment in growing oocyte [13].

### Motif finding analyses

Motif analyses were conducted using modules of the MEME suite [48] (version 4.9.0). DREME [17] was used for ab initio motif search, FIMO [49] was used for searching DNA sequences for motif occurrences, and TOMTOM [50] was used for finding matches between motifs and known recognition sites of DNA binding proteins.

### High throughput sequencing analyses

Percent methylation values of CGIs were taken from [12,13] for mouse GV and MII stage oocytes, and MeDIP-seq-derived log-transformed methylation fold-change values for mouse E8.5 embryos derived from Dnmt3L-deficient oocytes were taken from [14]. The complete annotation of CGIs with methylation data is part of the Additional file 4 and Additional file 5 (‘CGIs_methylation_Annotation’ spreadsheet).

The oocyte transcriptome was generated from analysing mRNA-seq data for growing (d10, [13]) and fully grown (d35, [13]; 7 to 8 week wild type and 7 to 15 week Dnmt3L-deficient [12]) oocytes using the Tuxedo protocol [22], including alignment with Tophat [51] (Bowtie-1 [52]), per-sample transcript reconstruction with Cufflinks [53] (v.2.0.1), and merging of per-sample reconstructed transcripts with Cuffmerge [22] (v.2.0.1). The complete annotation of CGIs with oocyte transcriptome data is part of the Additional file 4 and Additional file 5 (‘CGIs_transcripts_Annotation’ spreadsheet).

H3K4me3 ChIP-seq data for growing oocyte (d15, [13]) were reanalysed. ChIP-seq reads over CGIs and promoters of oocyte-expressed transcripts were counted using HTSeq (http://www-huber.embl.de/users/anders/HTSeq/). DESeq was used to normalise read counts for differences in sequencing depth between samples, to robustly estimate the variance of read counts between samples, and to variance-stabilise and log-transform the read count data [54]. Subsequently, regions enriched for H3K4me3 in the two IP samples compared to the inputs were identified from a linear model fitted with limma [55].

Cfp1 ChIP-seq data [29] were reanalysed using USeq [56] and MACS [57] with the default parameters. Since input DNA data were not available, we used the input samples for a CTCF ChIP-seq experiment in the same tissue (whole mouse brain) instead [58]. The sequence reads for the input samples were trimmed to be equal in length to the immunoprecipitated (IP) samples.

### CpG island classification

CGIs were classified into distinct classes related to their location relative to oocyte expressed transcripts for which the strand of origin could be determined by Cufflinks. Promoter-associated CGIs (PA) overlap a 1 kbp region (enriched in H3K4me3) around the TSS by at least 1 bp. Intragenic CGIs are located within a transcript, at least 1 kbp distant from the TSS. Distal intragenic CGIs overlap the 1 kbp region downstream from the end of the transcript. End-associated CGIs overlap the 1 kbp region around either the start or the end of a transcript that lacks strand information.

### CpG periodicity

The empirical distribution of the expected number of CpG pairs at distances from 0 to 1,000 bp was generated for each CGI from 1,000 independent permutations of its nucleotides while maintaining the original frequencies of all dinucleotides (dinucleotide frequency-invariant DNA sequence shuffling [25]). The CpG positions in each shuffled version of the sequence were recorded. From these positions, a pair-wise distance matrix was created. For each distance D from 0 to 1,000 or, if smaller, the length of the island L less two (the maximum distance between two CpGs in a sequence of length L is L-2), the number of CpG pairs was counted. For each distance D, the 1,000 counts generated from the 1,000 permutations of a sequence S form the empirical, expected distribution of the number of CpG pairs at distance D in S. Using this empirical distribution for S, the rank and corresponding empirical *P* value of the actually observed number of CpG pairs at distance D in S was determined. To test the significance of the number of CpG pairs at distances between 8 and 10 bp, the counts for these distances were added for each of the 1,000 permutations of S as well as for the original sequence S. The empirical p-value for this range of distances was then determined as above (the significance threshold was 0.05).

From the permutation-generated expected distributions, for each S and D, the expected number of CpG pairs in sequence S at distance D was derived by averaging the counts obtained from the 1,000 permutations of S. The expected number for S and D was used to normalise the number of actually observed CpG pairs at distance D in S, that is, observed over expected ratios (obs/exp) were generated. Finally, the obs/exp ratios for each distance D were averaged over all sequences in a CGI category with L - 2 ≥ D, where L is the length of the sequence, that is, excluding sequences that are too short to contain CpG pairs at distance D.

### Re-analysis of E2F1 ChIP-seq in MCF7 cells

SRA files with the E2F1 ChIP-seq (SRR167632-3) and input (SRR167638-40) reads were downloaded from the NCBI Short Read Archive, converted to FASTQ with fastq-dump from the SRA toolkit v2.3.5, and aligned to the GRCh37 (hg19) human reference genome using Novoalign v3.02.07. Calling of regions significantly enriched in the E2F1 ChIP-seq samples over input and identification of enriched DNA sequence motifs within those regions was performed using GEM v2.4.1 [59].

Promoter regions were derived from the transcripts annotated by UCSC Known Genes and, alternatively, by Gencode (comprehensive transcript set v19) as the regions from -1,500 bp to +500 bp of a TSS using Bash and Perl scripts and Bedops v2.4.2 [60]. Overlapping and abutting regions were merged. Each promoter region was then annotated with the maximum number of FANTOM5 CAGE tags per million mapped tags (TPM) that was observed for a CAGE tag cluster in MCF7 cells overlapping the promoter region [61].

Per CpG DNA methylation data for MCF7 cells generated by RRBS-seq were downloaded (GEO GSM683787 and GSM683793). Correlation between the two replicate samples was high (r^2^ = 97.97%) so that they were merged. CpGs with <10x coverage were discarded. Bedops was used to annotate each CGI from [10] with the number of assayed CpGs and their median percent methylation value.

The coordinates of occurrences of the CGCGC motif in the GRCh37 (hg19) genome were determined using dreg from the EMBOSS toolkit v6.6.0.

The above data set [see Additional file 7: BED format files, some with extra columns containing annotation] was the basis for overlap queries following filtering performed with bedops, followed by Fisher’s exact tests or logistic regression modelling in R. For example, TPM-annotated promoters were filtered by TPM >1 to generate the subset of expressed promoters. Similarly, CGIs were filtered by number of assayed CpGs >5, prior to determining their methylation state and testing or linear regression modelling.

## Abbreviations

CGIs: CpG islands; ESCs: embryonic stem cells; ICRs: imprinting control regions; MII: metaphase II; OR: odds ratio; RC: reverse complement; TPM: tags per million mapped tags; GV: germinal vesicle.

## Competing interests

The authors declare that they have no competing interests.

## Authors’ contributions

HS co-conceived and carried out most experiments and co-wrote the manuscript. RS conceived experiments, carried out some experiments and wrote the manuscript. Both authors read and approved the final manuscript.

## Supplementary Material

Additional file 1 Supplementary Tables. Click here for file

Additional file 2 Supplementary Figures. Click here for file

Additional file 3 Supplementary Results and Methods. Click here for file

Additional file 4 Description of supplementary spreadsheets. Click here for file

Additional file 5 Supplementary spreadsheets. Click here for file

Additional file 6 Mini-website with GEM peak calling and motif analysis results for E2F1 ChIP-seq in MCF7 cells. Click here for file

Additional file 7 BED files with data underlying the statistical analyses of the E2F1 ChIP-seq peaks in MCF7 cells; coordinates are for the hg19 human reference genome build. Click here for file
