# Supplementary material for: Protection of CpG islands against de novo DNA methylation during oogenesis is associated with the recognition site of E2f1 and E2f2
Source: Epigenetics Chromatin. 2014 Oct 21;7:26. doi: 10.1186/1756-8935-7-26 (PMC4255709; doi:10.1186/1756-8935-7-26)
Supplement: Additional file 1 — Supplementary Tables. [file 1756-8935-7-26-S1.pdf]

Table S1. Genomic coordinates of the 23 permanent maternal gDMRs The coordinates are from (14) (boundaries of significantly 5mC-enriched regions by MeDIP-seq). Kelsey ID: CGI identifiers from (13). These CGIs overlap the permanent maternal gDMRs and are methylated in at least one of the GV and MII oocyte samples from (13) or in the wildtype oocyte sample from (12).

Chromosome	Start	End	gDMR Name	#Occurrences of TGCCGC	Kelsey CGI ID
**chr10**	12808823	12812542	*Zac1*	5	#10-56
**chr11**	11924753	11927373	*Grb10*	3	#11-133
**chr11**	22871199	22874744	*U2af1-Rs1*	2	#11-189
**chr13**	47106262	47106763	*AK008011*	4	#13-310
**chr15**	72638946	72641843	*Peg13*	7	#15-248
**chr15**	96885270	96886284	*Slc38a4*	0	#15-703
**chr17**	87524084	87525819	*Socs5*	4	#17-1073
**chr17**	12933529	12936244	*Igf2r/Air*	8	#17-72
**chr18**	13130394	13133109	*Impact*	5	#18-68
**chr2**	152511620	152513915	*Mcts2*	3	#2-1310
**chr2**	157384230	157387951	*Nnat*	7	#2-1428<br>#2-1429
**chr2**	174119695	174127298	*Nespas*	13	#2-1709<br>#2-1710<br>#2-1711
**chr2**	174151206	174155152	*Gnas-exon1a*	3	#2-1712
**chr6**	30684874	30689459	*Mest*	11	#6-140<br>#6-141<br>#6-142
**chr6**	47974007	47975979	*Zfp777*	3	#6-242
**chr6**	58855970	58857759	*Nap1l5*	5	#6-371
**chr6**	4696114	4698664	*Peg10*	4	#6-8
**chr7**	6681534	6684030	*Zim2*	7	#7-105
**chr7**	135830911	135832795	*Inpp5fv2*	6	#7-1597
**chr7**	150480004	150483196	*Kcnq1ot1*	4	#7-1779
**chr7**	6083480	6084890	*Zfp787*	3	#7-86
**chr7**	67148934	67150346	*Snurf/Snrpn*	10	#7-980
**chr8**	125387861	125390344	*Cdh15*	2	#8-1221

Table S2. Summary of permanent maternal gDMR CpG periodicity analyses. DMR CGIs have the name of the respective gDMR, followed by the length of the CGI in cases of multiple CGIs belonging to the same gDMR. The second column marks the DMR CGIs with significant (empirical $p< 0.05$) observed numbers of pairs of CpGs at distances between 8 and 10bp. The third column marks the DMR CGIs whose obs/exp ratio noticeably peaks at 8-10bp. DMR CGIs in bold red additionally show a periodic pattern of peaks. The forth column marks the DMR CGIs with a dominant period between 8 and 10bp by the ACC method, or the length of the dominant period, if distinct from 8-10bp. The fifth column marks the DMR CGIs that contain a peak within the 100-125Hz (8-10bp) range with an amplitude >0.07 by the FFT method. In the DMR CGIs marked in bold red, the 8-10bp period is dominant.

DMR CGI	Significant number of CpG pairs at 8-10bp	obs/exp peaks at 8-10bp (red: periodic peaks)	ACC peaks at 8-10bp	FFT spectrum peaks at 100-125Hz (amplitude >0.07; red: no other more powerful frequency)
Ak008011			Y	**Y**
Cdh15			5-6	
Gnas-exon1a				Y
Grb10	Y	Y		
Igf2r/Air	Y	Y	Y	Y
Impact	Y	**Y**	Y (5)	Y
Inpp5fv2				Y
Kcnq1ot1				Y
Mcts2				
Mest_426			Y	**Y**
Mest_452	Y	**Y**		
Mest_742				
Nap1l5				Y
Nespas_1348	Y	Y	Y	Y
Nespas_2795				
Nespas_463			12-14	
Nnat_239			4-5	
Nnat_621		**Y**	Y	**Y**
Peg10	Y	Y	7	
Peg13	Y	**Y**	Y	Y
Slc38a4		**Y**	7	
Snurf/Snrpn				Y
Socs5	Y	**Y**	Y	Y

**U2af1-rs1**	Y	**Y**	Y	**Y**	
**Zac1**		Y		**Y**	
**Zfp777**					
**Zfp787**		Y	6-7		
**Zim2**	Y	**Y**	Y	**Y**	

Table S3. Pair-wise Pearson correlation coefficients between factors and between each factor and the CGI methylation state. Each factor as well as the Methylation State are binary-valued vectors of length equal to the number of studied CGIs. Methylated CGIs are encoded using the value one. A value of one for each of the other factors encodes the presence of the respective CGI feature. Consequently and as expected, all factors are negatively correlated with CGI methylation. The largest correlation (0.62) between any two factors was observed for the CGI being associated with an oocyte-active promoter (PA) and the CGI having R-loop formation potential (R-loop).

	PA	Methylation State	CGCGC motif	Cfp1	R-loop	H3K4me3
**PA**	1	-0.24	0.17	0.45	0.62	0.31
**Methylation State**		1	-0.28	-0.27	-0.04	-0.29
**CGCGC motif**			1	0.21	0.07	0.13
**Cfp1**				1	0.29	0.22
**R-loop**					1	0.18
**H3K4me3**						1

Table S4. Fitted logistic linear regression models for hypomethylation-associated factors, and test results. The test of the second model versus the first (null) model (columns 4-6) determines for each factor in isolation, whether it has significant power to predict the CGI methylation state. The third model versus the second model test (columns 7-9) determines whether adding the CGCGC motif to another factor significantly improves the prediction of the CGI methylation state, i.e., whether CGCGC has significant (linearly) independent predictive power Df: Degrees of freedom. The coefficient values of the fitted models are shown alongside with the factors. All coefficients were significantly different from zero (t-test; $p < 10^{-13}$) except for the Rloop coefficient in the third model (p= 0.0515).

First model	Second model	Third model	Reduction in deviance	Df	Chi-square p-value	Reduction in deviance	Df	Chi-square p-value
M = -2	M = -1.29 - 2.46*H3K4me3	M = -0.39 - 2.36*H3K4me3 -1.57*Motif	851.57	1	0	483.53	1	0
M = -2	M = -0.98 - 1.69*Motif	/	609.51	1	0	/	/	/
M = -2	M = -1.16 - 1.65*Cfp1	M = -0.42 - 1.42*Cfp1 - 1.45* Motif	583.73	1	0	415.61	1	0
M = -2	M = -1.32 - 1.57*PA	M = -0.50 - 1.38*PA - 1.52*Motif	498.13	1	0	464.65	1	0
M = -2	M = -1.90 - 0.26*Rloop	M = -0.95 - 0.14*Rloop - 1.72*Motif	14.054	1	1.78e-04	598.80	1	0

Table S5. Test results for pair-wise interactions between hypomethylation- associated factors. Df: Degrees of freedom. Chi-square p-values < 0.05 are highlighted in red.

First model	Second model	Reduction in deviance	Df	Chi-square p-value
**PA + Motif**	PA + Motif + PA:Motif	1.3542	1	0.24
**PA + Rloop**	PA + Rloop + PA: Rloop	233.41	1	**0**
**PA + H3K4me3**	PA + H3K4me3+ PA: H3K4me3	1.1127	1	0.29
**PA + Cfp1**	PA + Cfp1+ PA: Cfp1	24.719	1	**6.63e-07**
**Motif + Rloop**	Motif + Rloop + Motif: Rloop	2.3578	1	0.12
**Motif + H3K4me3**	Motif + H3K4me3+ Motif: H3K4me3	1.3453	1	0.25
**Motif + Cfp1**	Motif + Cfp1+ Motif: Cfp1	14.148	1	**1.69e-4**
**H3K4me3+ Rloop**	H3K4me3+ Rloop + H3K4me3: Rloop	2.66	1	0.10
**H3K4me3+ Cfp1**	H3K4me3+ Cfp1+ H3K4me3: Cfp1	0.66337	1	0.42
**Rloop + Cfp1**	Rloop + Cfp1+ Rloop: Cfp1	50.527	1	**1.18e-12**
