# Supplementary material for: Protection of CpG islands against de novo DNA methylation during oogenesis is associated with the recognition site of E2f1 and E2f2
Source: Epigenetics Chromatin. 2014 Oct 21;7:26. doi: 10.1186/1756-8935-7-26 (PMC4255709; doi:10.1186/1756-8935-7-26)
Supplement: Additional file 2 — Supplementary Figures. [file 1756-8935-7-26-S2.pdf]

Figure S1

A

	Motif	Logo	E-value
1	CCACAGDG	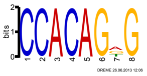	3.0e-007
2	TGKGGCCW	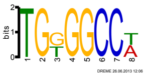	1.2e-006
3	CACWGCCA	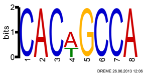	1.4e-004
4	GGRCAGCA	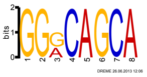	2.8e-003
5	CWGCCCCA	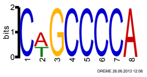	6.7e-003
6	CAGGGVCA	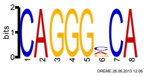	7.1e-003
7	CYACCAGC	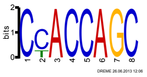	1.7e-002
8	ACSCATGG	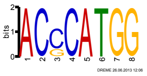	2.0e-002
9	CCCTATRC	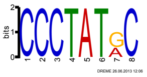	2.0e-002
10	CCTGTGGK	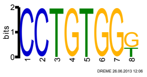	3.3e-002
11	GGCACAGR	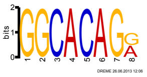	3.3e-002
12	AGGCCMTG	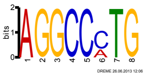	3.8e-002

B

	Motif	Logo	E-value
1	CAGAGCCM	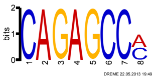	5.5e-004
2	GRCACCYC	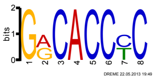	3.8e-003
3	CCCAGRGC	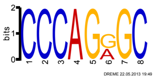	1.9e-002
4	AGGKCCAG	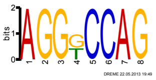	2.7e-002
5	GGGGCTSA	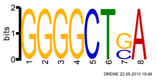	4.0e-002
6	CACCCRCC	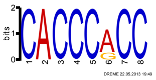	4.2e-002

Figure S1. **Ab initio motif search in oocyte-methylated CGIs and promoters upstream of oocyte-methylated intragenic CGIs**. (A) DREME results for the comparison of methylated CGIs (n = 988) with unmethylated CGIs (n = 7,372), including +/- 1kbp shores. (B) DREME results for oocyte-active promoters with methylated CGIs (n= 103) versus oocyte-active promoters without methylated CGIs (n= 2,017) comparison (promoter regions of +/- 4kbp around the TSS). Any overlapping sequences between the two compared sets were excluded and overlapping sequences within each set were merged. Other terms are analogous to those in Figure 2.A.

Figure S2

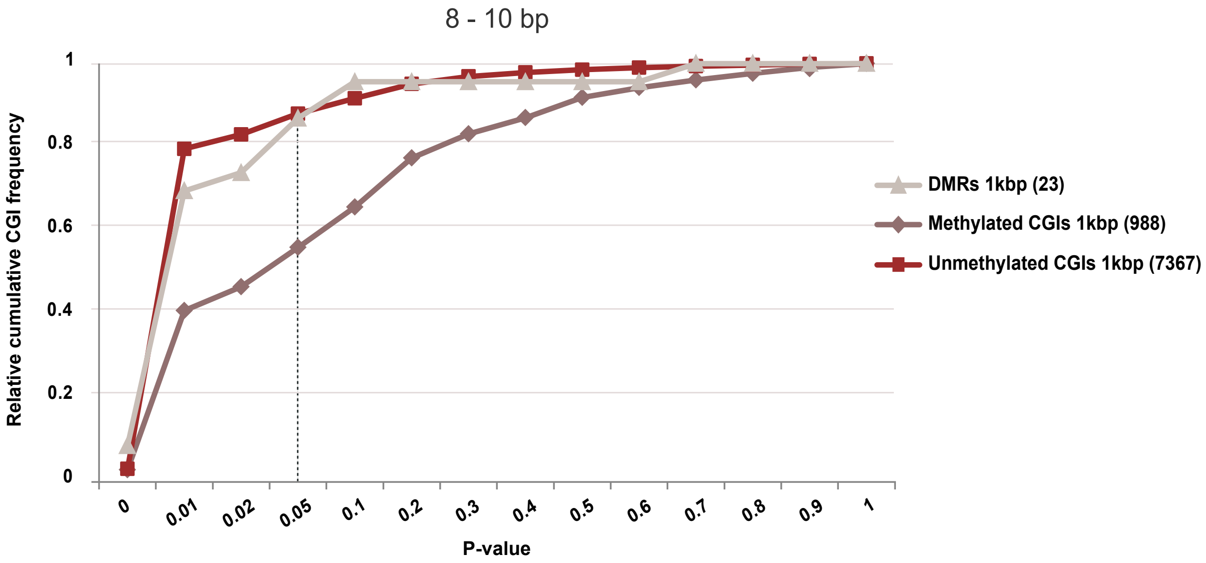

Figure S2. **Cumulative empirical p-value distribution**. The distribution for the 8-10bp distance range is shown for CGIs including shores (+/- 1kbp flanking sequence). Other terms are analogous to those in Figure 4.

Figure S3

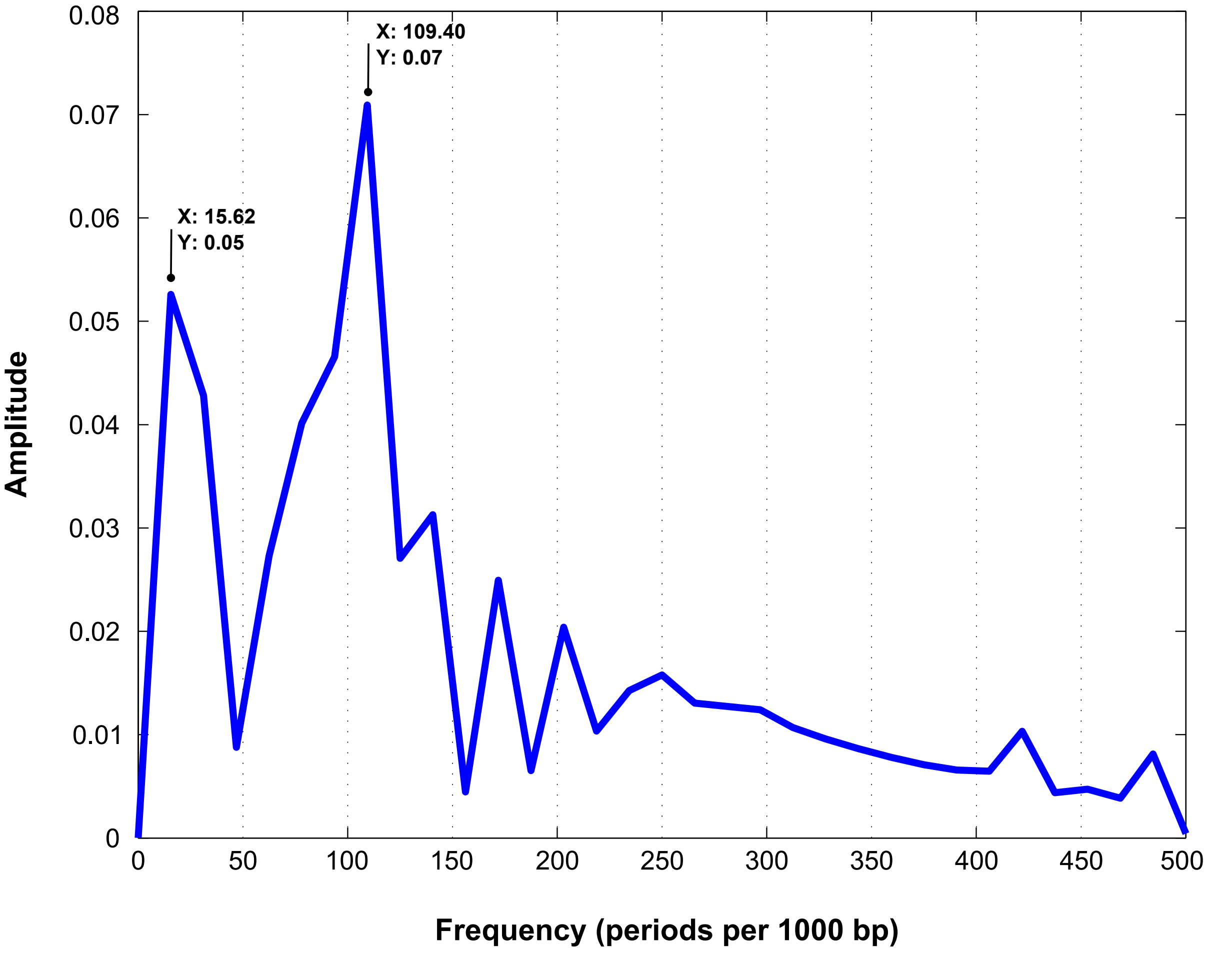

Figure S3. **Average frequency spectrum for the 28 CGIs in permanent maternal gDMRs**. The frequency spectrum was calculated from the smoothed and linearly de-trended obs/exp ratios for distances between 5 to 68bp. The spectrum is a plot of frequency in ‘Hz’ (periods per 1000bp) along the x-axis versus the amplitude (power) along the y-axis. A period length of between 8 and 10bp corresponds to a frequency of between 125 and 100 ‘Hz’ (periods per 1000 bp). The two frequencies with the greatest power are highlighted: 15.62Hz (64bp period) and 109.40Hz (9.14bp period). The 64bp period was neglected since it is an artefact of the analysis having been performed on the obs/exp data for 64bp.

Figure S4

A

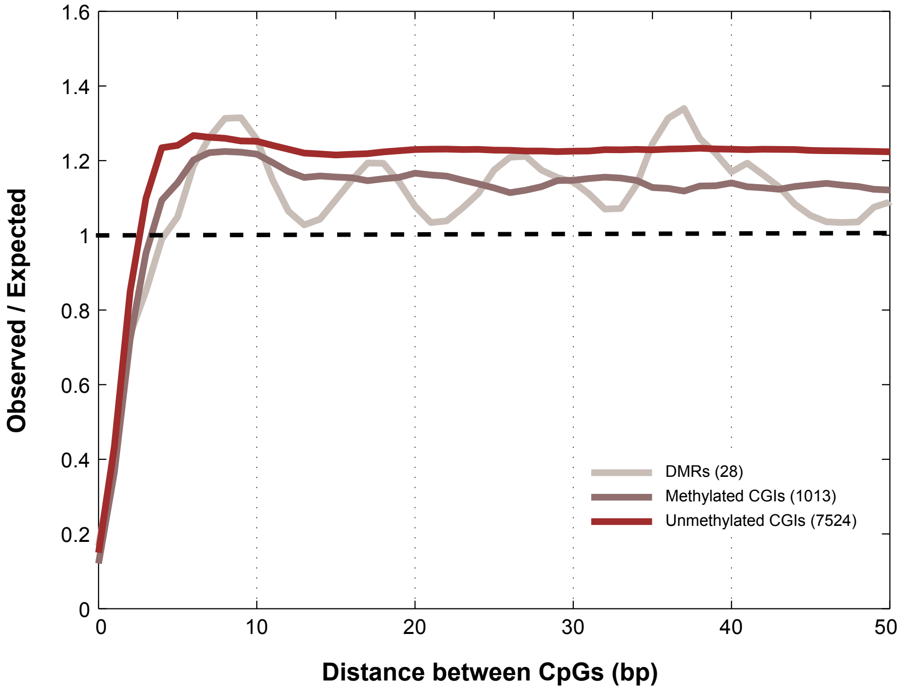

B

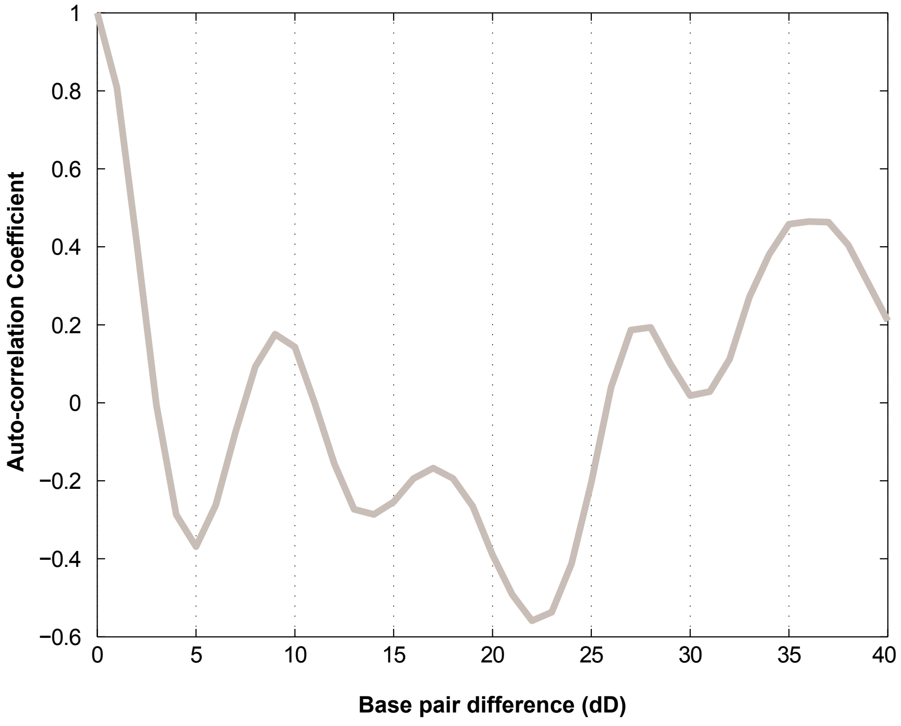

Figure S4. **CpG periodicity analysis via observed/expected ratios and auto-correlation**. (A) Average obs/exp ratios for distances up to 50bp per CGI category. Other terms are analogous to those in Figure 3. (B) Auto-correlation coefficients derived from the smoothed and de-trended obs/exp ratios for distances between 5 and 45bp versus for distances between 5+ dD and 45+ dD bp for dD between 0 and 40, for the DMR CGIs (n= 28). DMRs: maternal permanent gDMR CGIs.

Figure S5 part 1

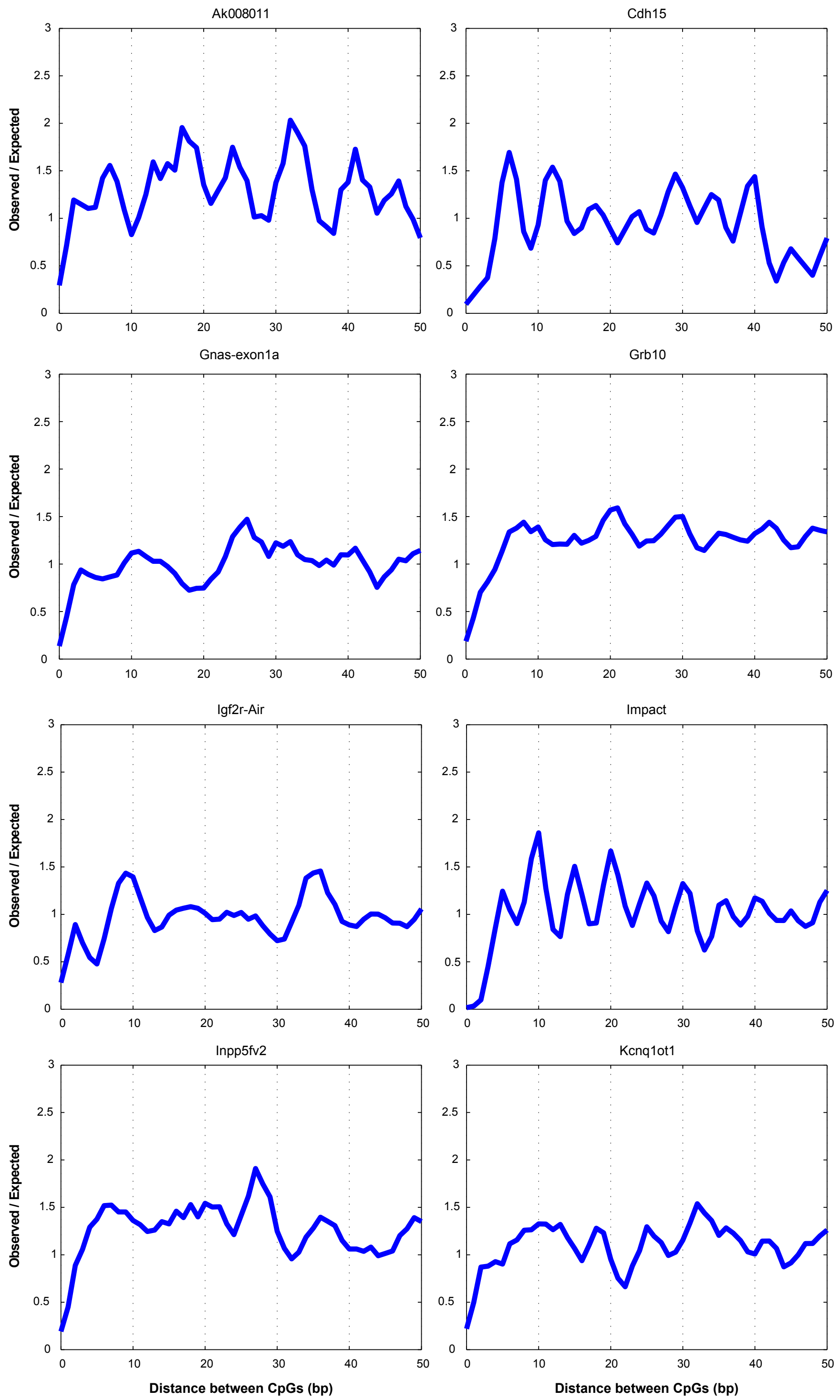

Figure S5 part 2

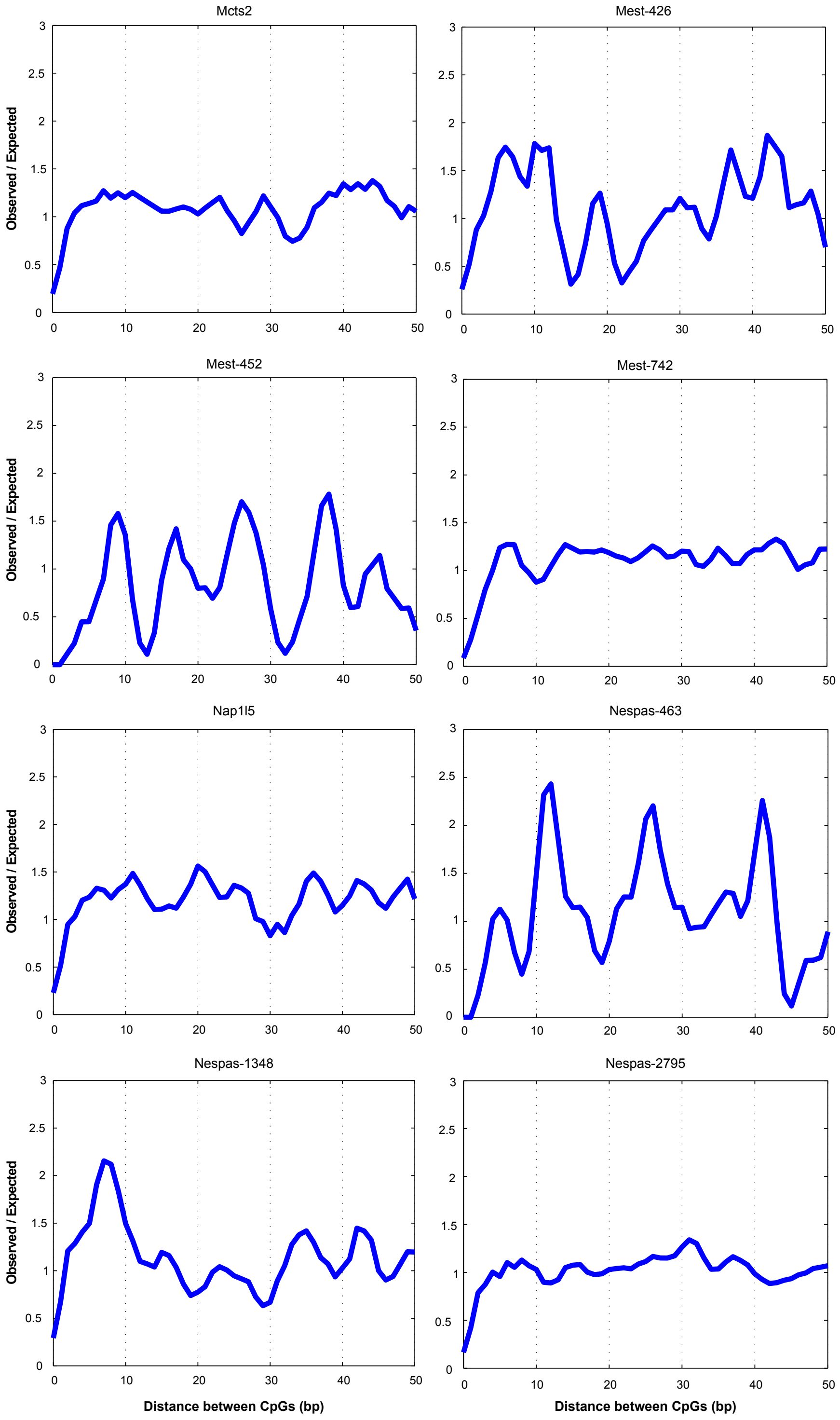

Figure S5 part 3

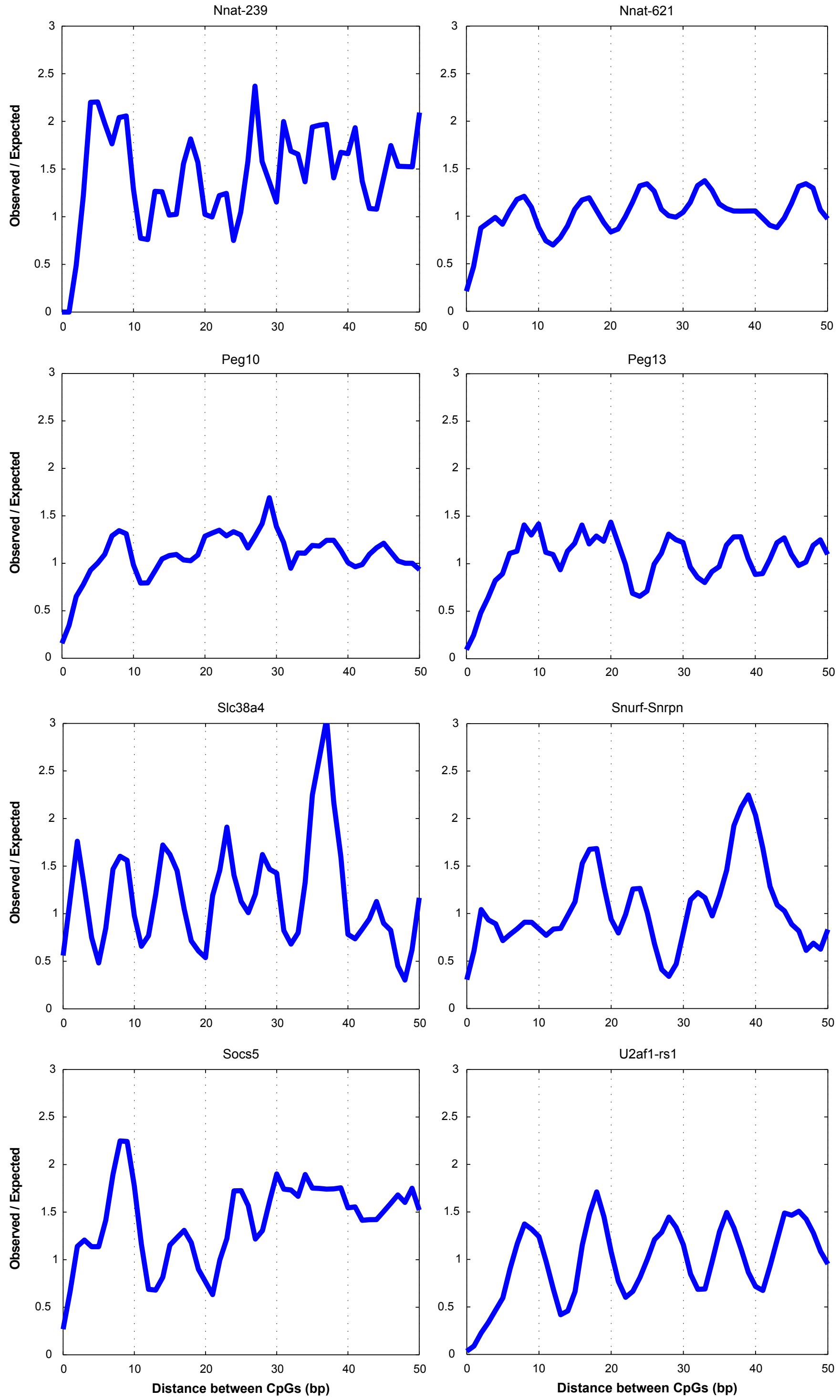

Figure S5 part 4

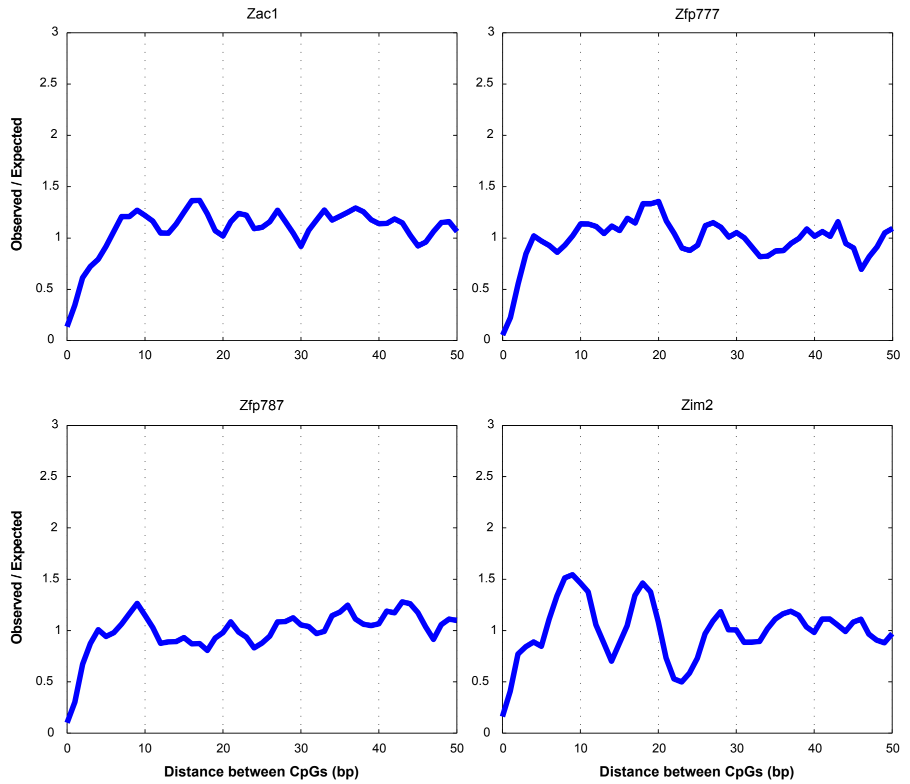

Figure S5. **Observed/expected ratios for individual DMR CGIs**. The observed/expected ratio was calculated for distances up to 50bp. The DMR CGIs are AK008011, Cdh15, Gnas-exon1a, Grb10, Igf2r-Air, Impact, Inpp5f, Kcnq1ot1, Mcts2, Mest-426, Mest-452, Mest-742, Nap1l5, Nespas-463, Nespas-1348, Nespas-2795, Nnat-239, Nnat-621, Peg10, Peg13, Slc38a4, Snrpn-Snurf, Socs5, U2af1-rs1 Zac1, Zfp777, Zfp787, and Zim2. The numbers appended to some DMR CGI names indicate the length of the CGI and are used to uniquely identify them.

Figure S6 part 1

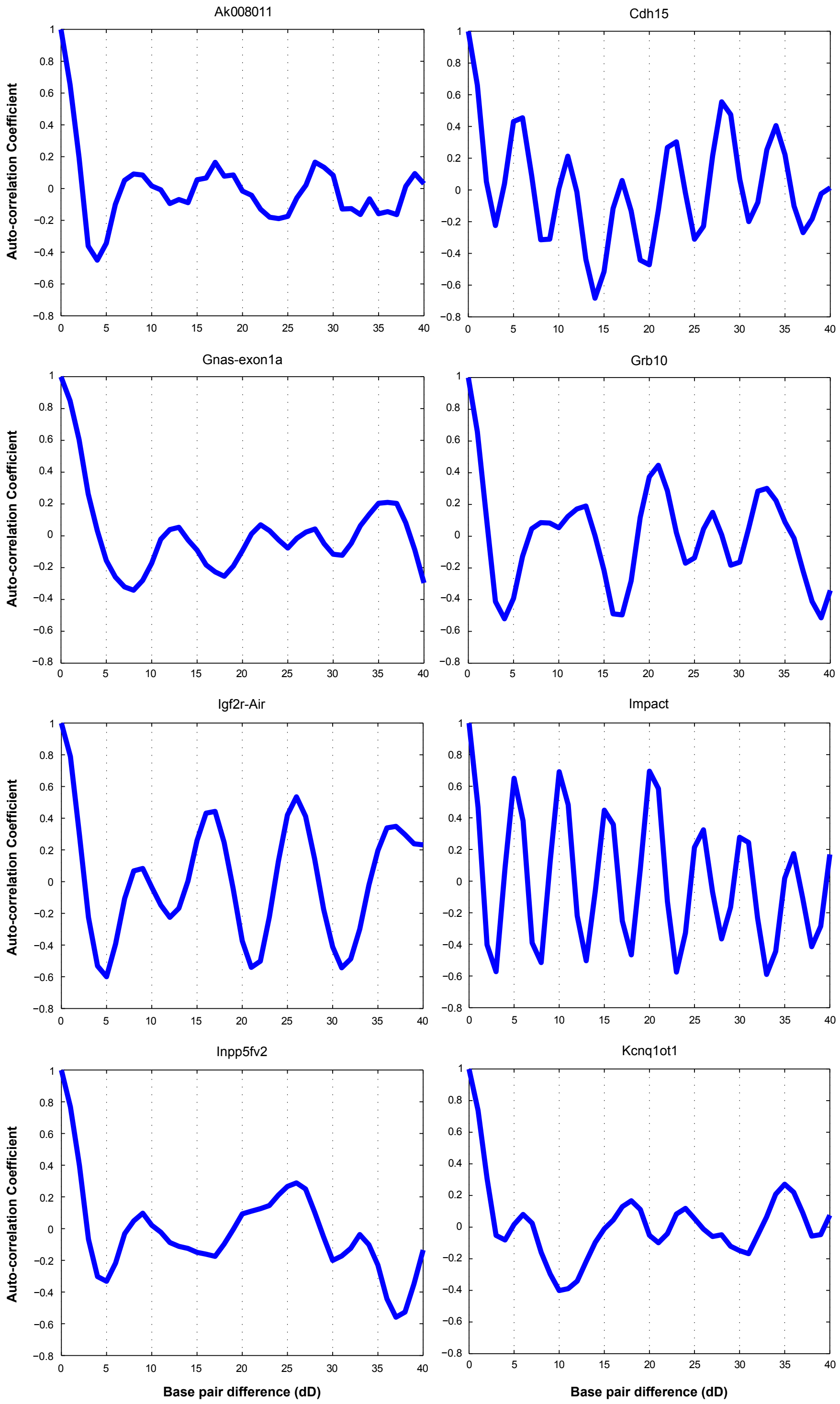

Figure S6 part 2

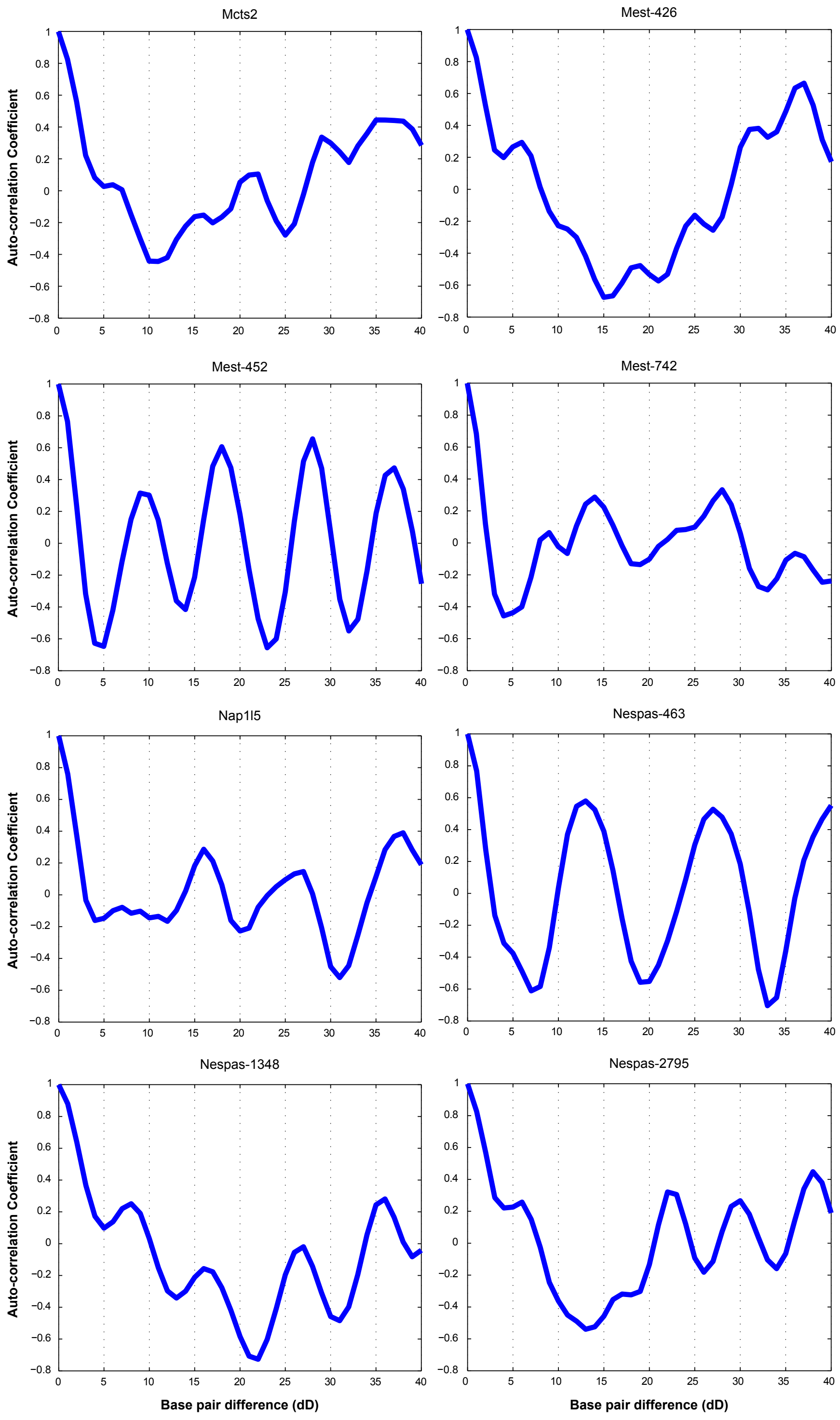

Figure S6 part 3

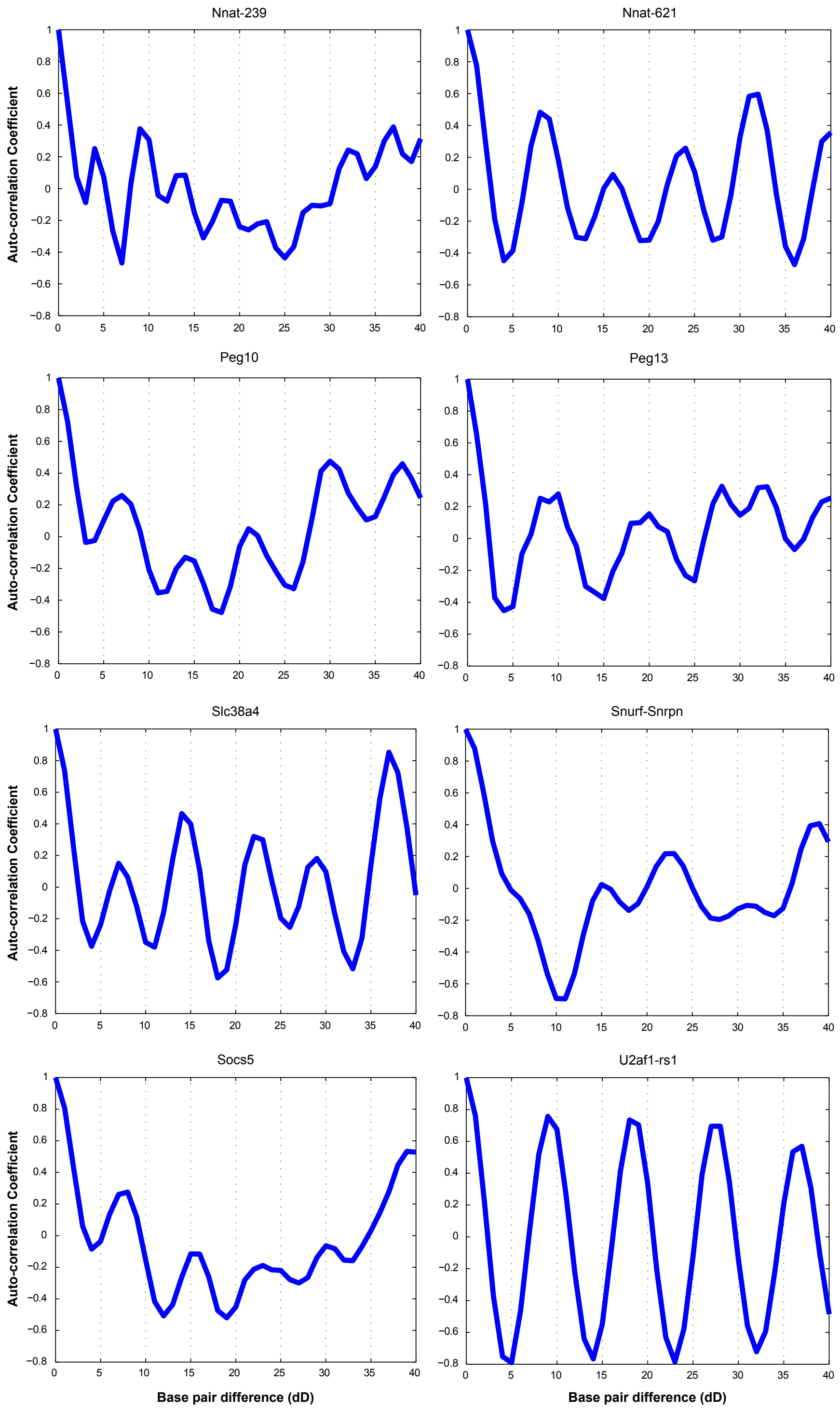

Figure S6 part 4

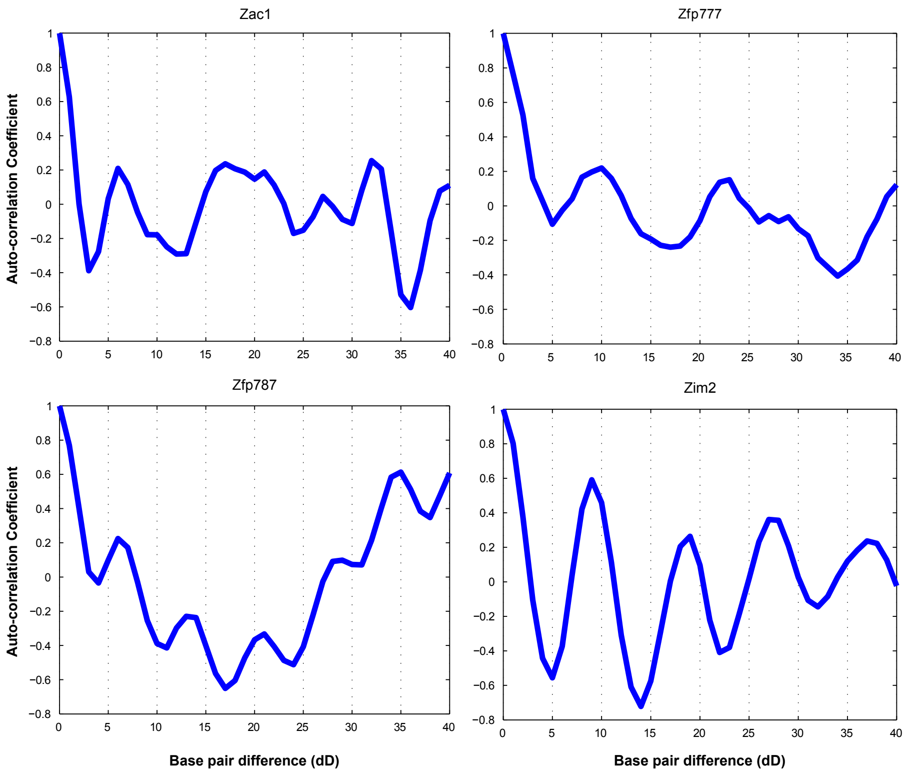

Figure S6. **Auto-correlation coefficients for individual DMR CGIs**. The ACC was calculated from the smoothed and de-trended obs/exp ratios for distances between 5 and 45bp versus for distances between 5+ dD and 45+ dD bp for dD between 0 and 40. The order of the DMR CGIs is analogous to Figure S5.

Figure S7 part 1

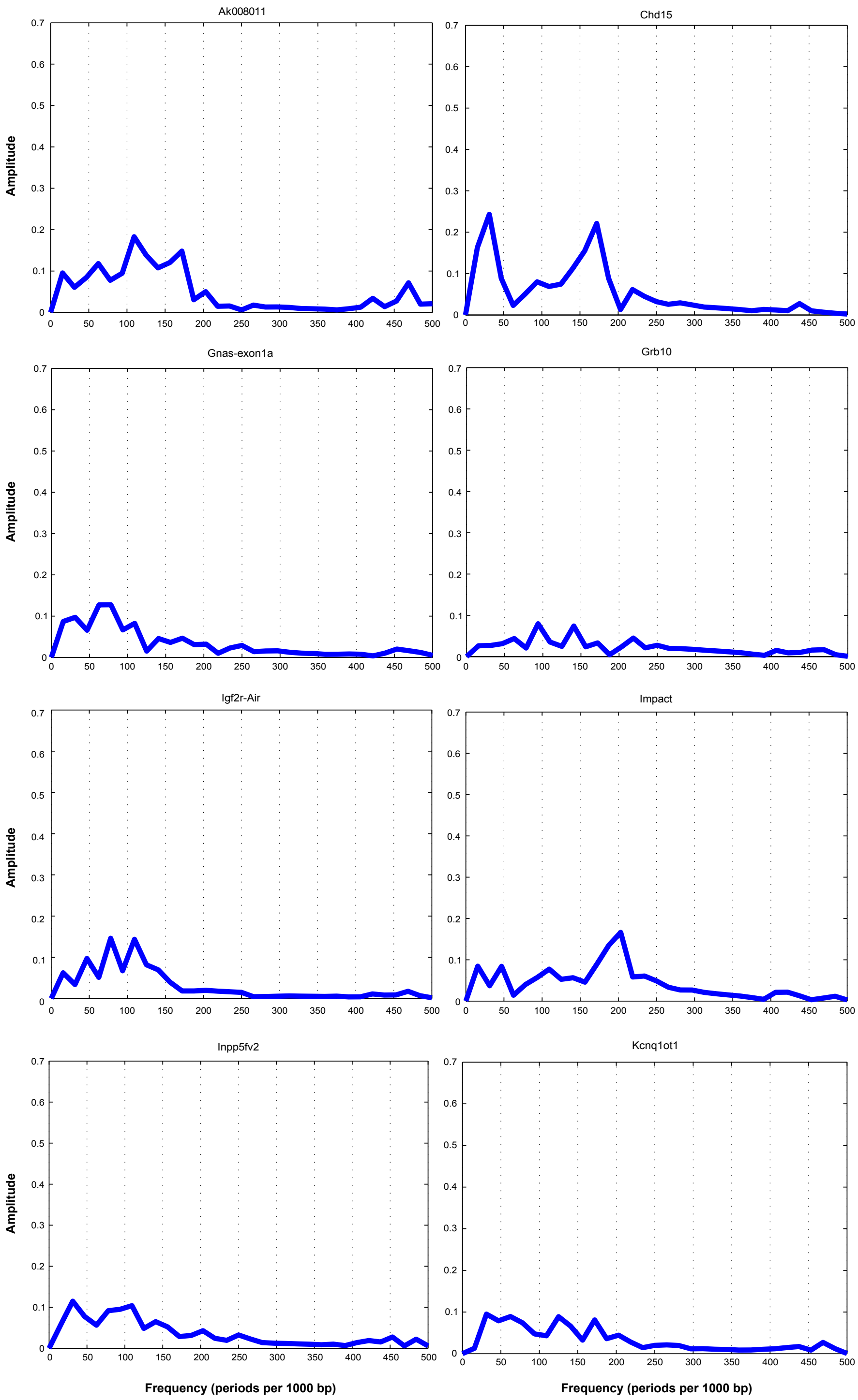

Figure S7 part 2

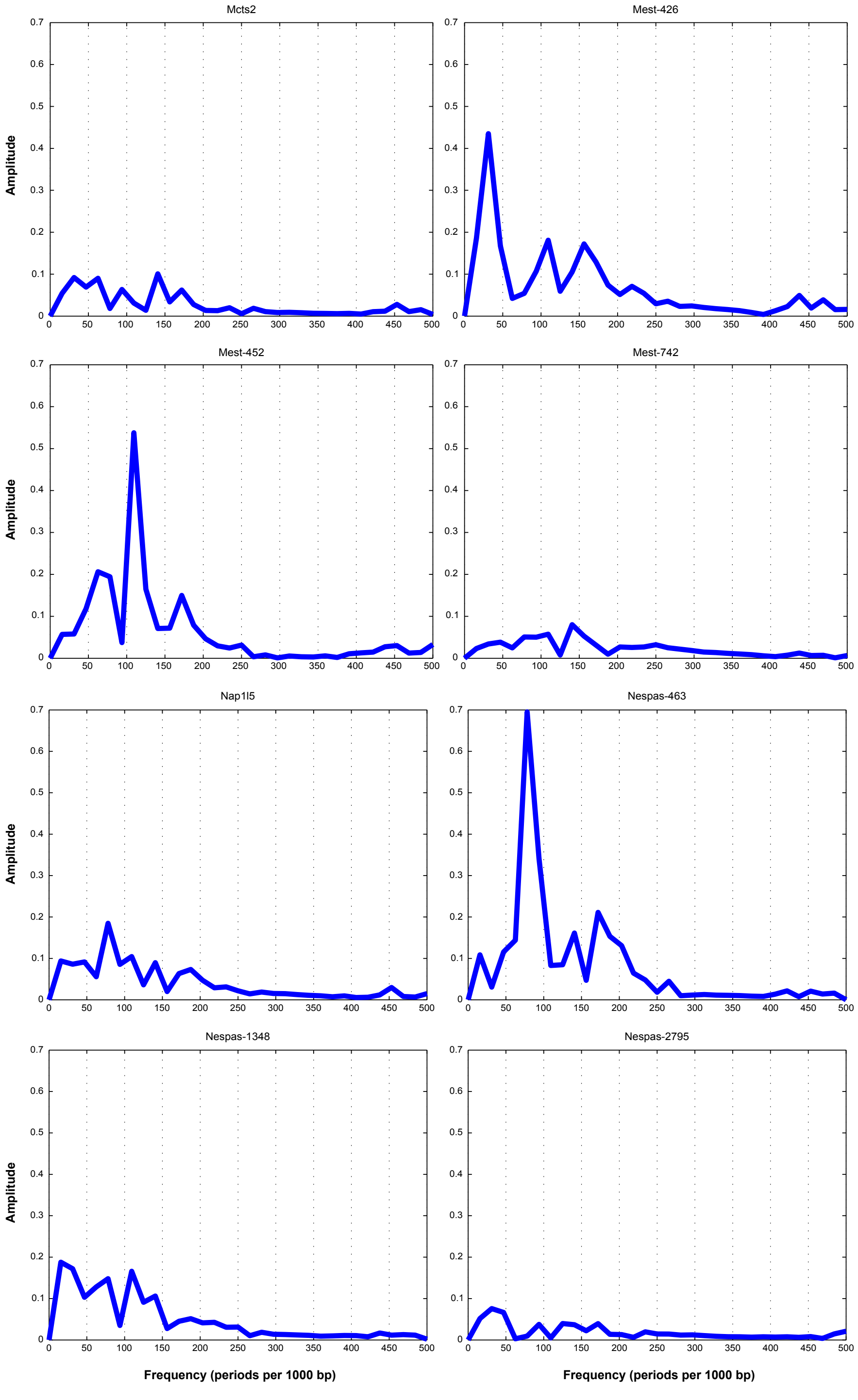

Figure S7 part 3

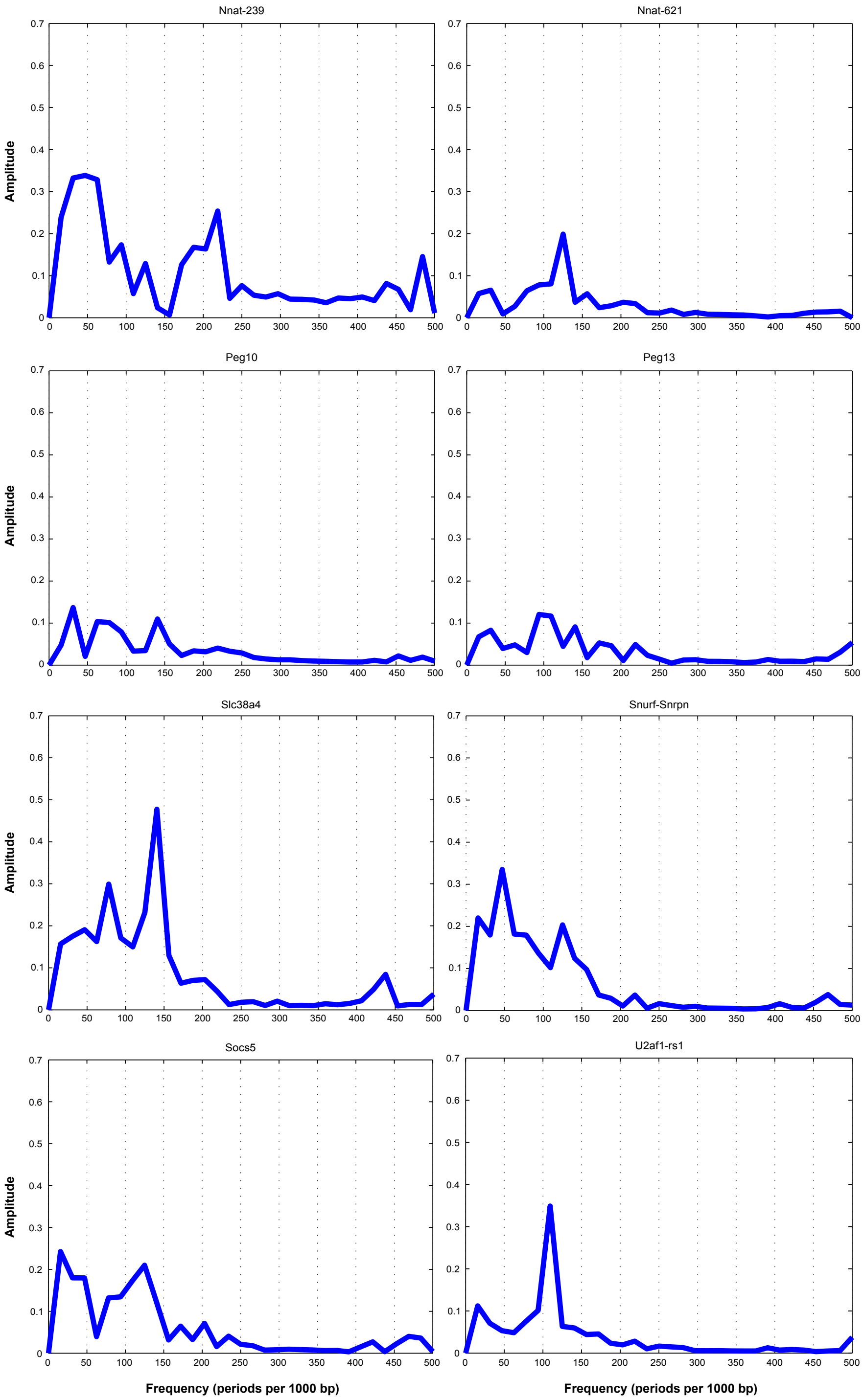

Figure S7 part 4

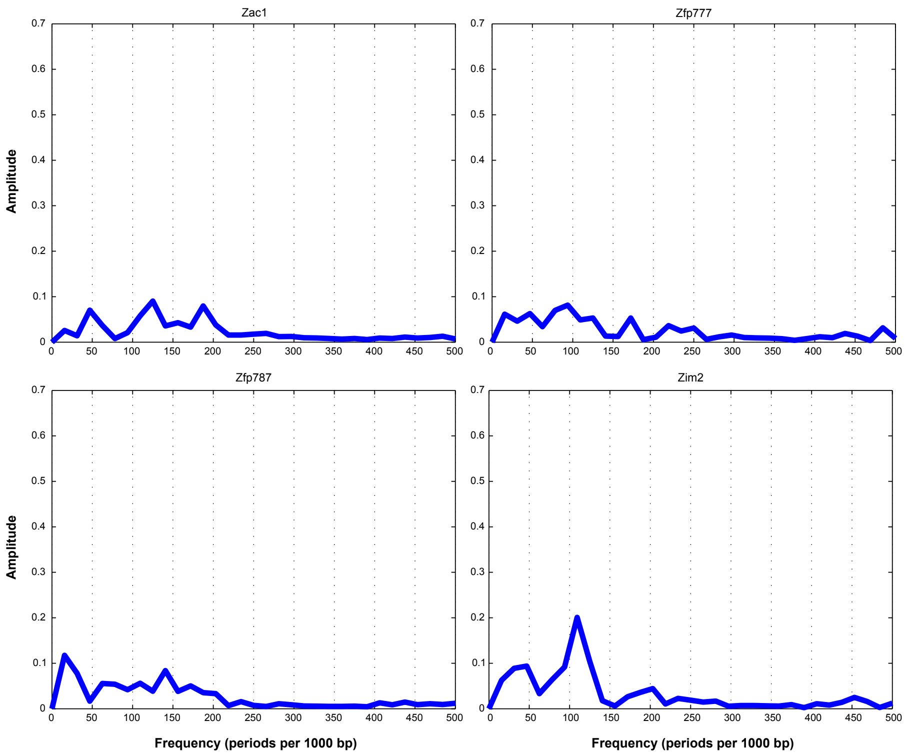

Figure S7. **Frequency spectra for individual DMR CGIs**. The order of the DMR CGIs is analogous to Figure S5.

Figure S8

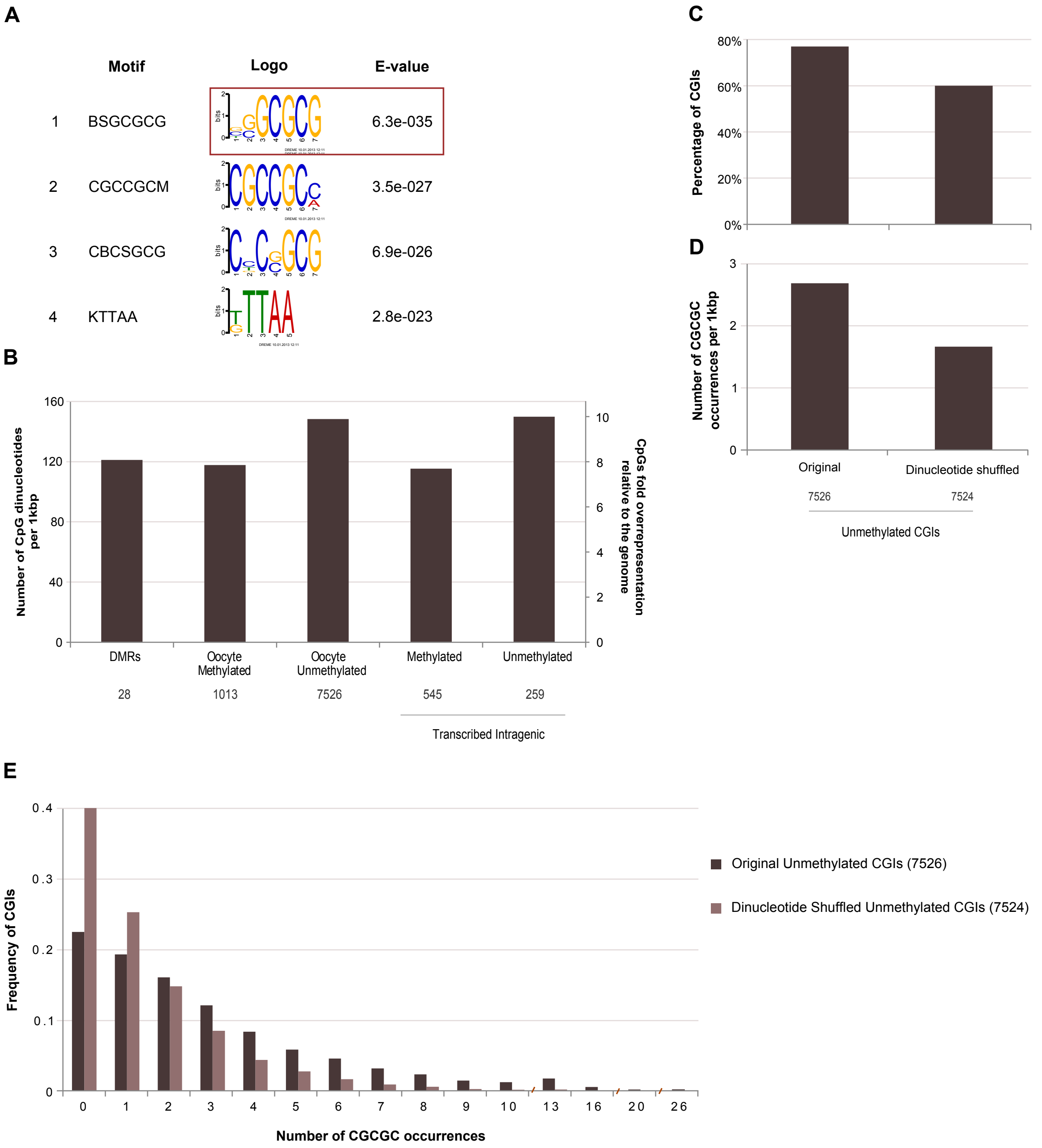

Figure S8. **Supplemental CGCGC motif analyses**. (A) DREME-reported motifs for intragenic unmethylated CGIs compared to intragenic methylated CGIs after masking the repeats. The highlighted, most significantly enriched motif, CGCGCS when reverse-complemented (DREME searches are not strand-specific), is almost identical to the originally identified motif MCGCGCS. (B) Density of CpGs per CGI category. The overall density pattern is similar to that of the CGCGC motif (Figure 6.D). Presence (C) and density per 1kbp (D) of the CGCGC motif in unmethylated CGIs versus unmethylated but sequence-shuffled CGIs. The y-axis in (C) represents the absolute percentage of CGIs containing at least one occurrence of the motif. The y-axis in (D) represents the number of motif occurrences per 1kbp. The difference between unmethylated and shuffled unmethylated CGIs in both figures is significant ($p < 2.2$ e-16; Fisher's exact test). (E) Relative distribution of the number of occurrences (x-axis) of the CGCGC motif in unmethylated CGIs (dark brown) and in sequence-shuffled unmethylated CGIs with identical CpG densities (pink). The number between parentheses shows the total number of sequences. Two unmethylated CGI sequences were excluded before shuffling because they contain ambiguous nucleotide codes. DMRs: maternal permanent gDMR CGIs.

Figure S9

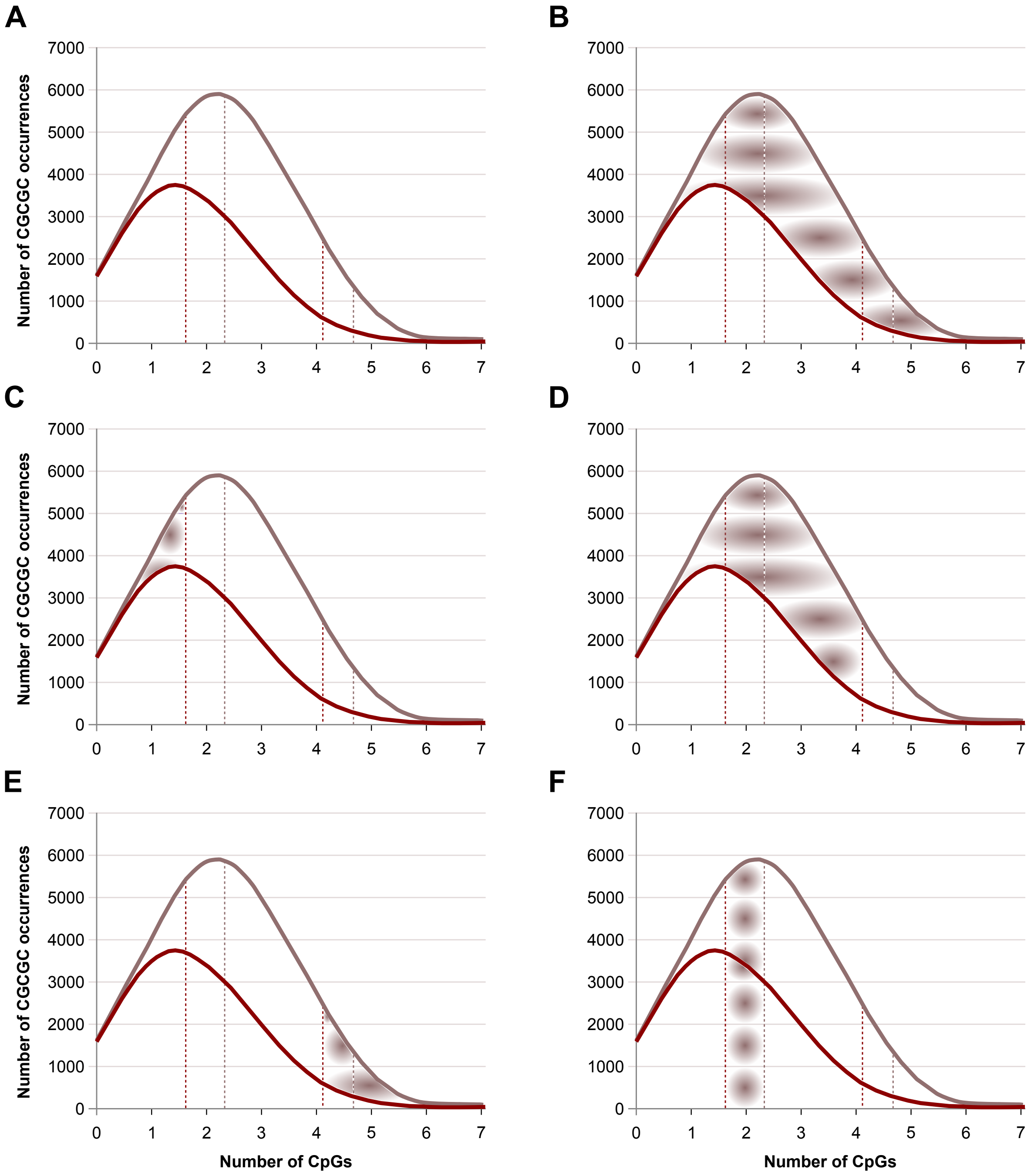

Figure S9. **Number of motif occurrences as a function of the number of CpGs in the +/- 10bp flanking region of the motif (excluding CpGs in other motif occurrences) for the original (light brown) and the shuffled (red) unmethylated sequences**. The x-axis represents the number of CpGs in the flanking region and the y-axis represents the number of motif occurrences. The left vertical light brown line marks the average of the original set of sequences, while the left red line marks the average for the shuffled set, i.e., the value expected by chance. The shorter lines to the right mark the upper bound of the 95% confidence intervals for the two distributions. There are 8,755 extra motif occurrences in the original relative to the shuffled sequences (B). Of those, 644 have fewer CpGs in their flanking regions than expected by chance, i.e., fewer than the average of the (null) distribution for the shuffled sequences (C). They in particular cannot be explained by locally high CpG density. In addition, most of the extra occurrences (8,317) in the original sequences have a local CpG content within the 95% confidence interval of the expected value (average of the null distribution) (D). Only 5% (438) of the extra motif occurrences in the original unmethylated sequences have a significantly greater than expected local CpG density (E). Overall, the difference between the means of the two distributions corresponds to less than one CpG (F).

Figure S10

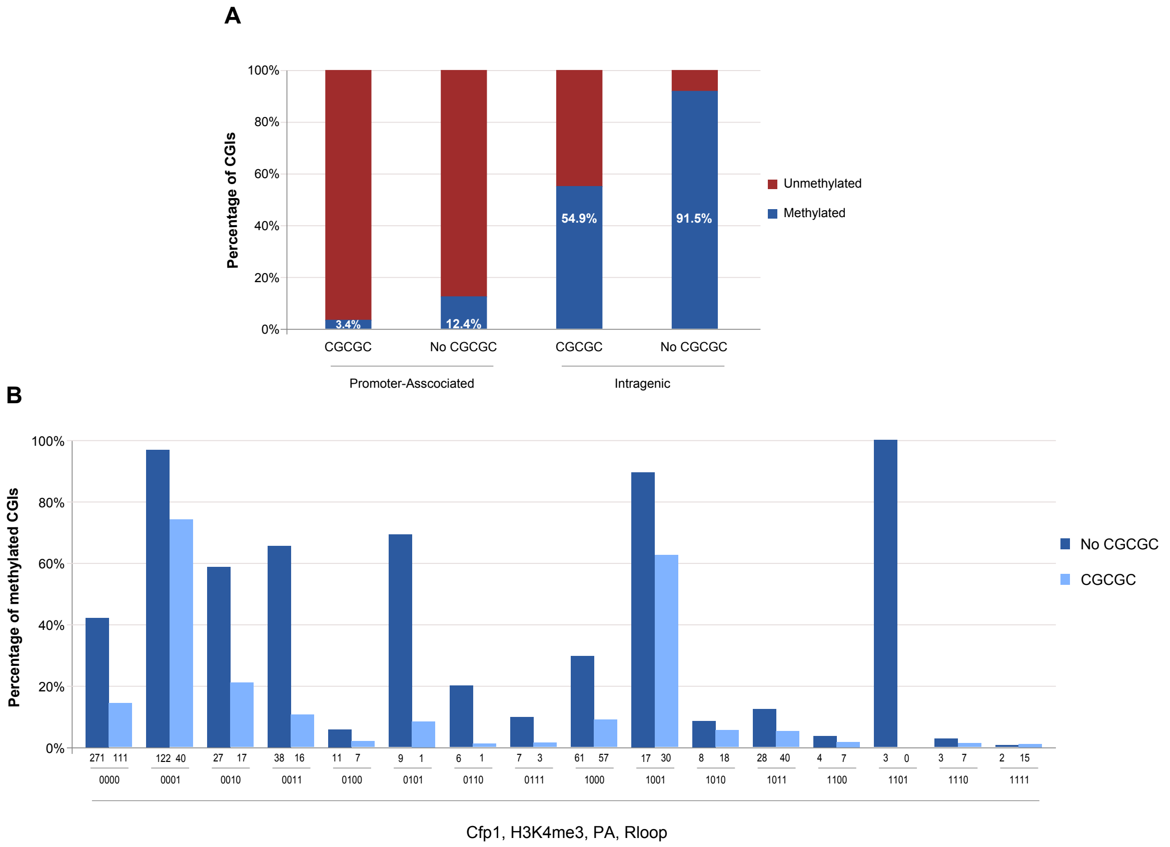

Figure S10. **Effect of the CGCGC motif and combinations of factors on the fraction of methylated CGIs**. (A) Effect of the presence versus absence of the CGCGC motif on the ratio of methylated (blue) versus unmethylated (red) CGIs, separately for the promoter-associated and intra-genic CGI categories. (B) Effect of the presence (light blue) versus absence (dark blue) of the CGCGC motif on the fraction of methylated CGIs, separately for sixteen CGI categories, each defined by a combination of the other four factors previously associated with CGI hypo-methylation (Cfp1 binding, H3K4me3, promoter activity in the oocyte, R-loop formation potential). The numbers underneath each bar represent the total number of CGIs.

Figure S11

A

**Name**	MA0375.1
**Alt. Name**	RSC30
**Database**	JASPAR_CORE_2009_.meme
**p-value**	3.36362e-06
**E-value**	0.00289944
**q-value**	0.00489276
**Overlap**	7
**Offset**	1
**Orientation**	Normal

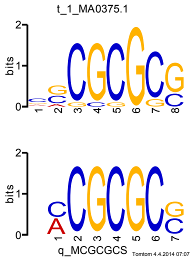

**Name**	MA0374.1
**Alt. Name**	RSC3
**Database**	JASPAR_CORE_2009_.meme
**p-value**	5.73393e-06
**E-value**	0.00494265
**q-value**	0.00489276
**Overlap**	6
**Offset**	-1
**Orientation**	Normal

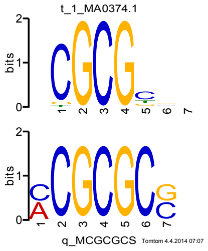

B

**Name**	MA0024.1
**Alt. Name**	E2F1
**Database**	JASPAR_CORE_2009_.meme
**p-value**	0.000909822
**E-value**	0.784266
**q-value**	0.154898
**Overlap**	5
**Offset**	-2
**Orientation**	Reverse Complement

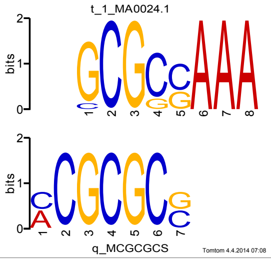

**Name**	UP00065_1
**Alt. Name**	Zfp161_primary
**Database**	uniprobe_mouse.meme
**p-value**	0.00093696
**E-value**	0.803984
**q-value**	0.154898
**Overlap**	7
**Offset**	6
**Orientation**	Reverse Complement

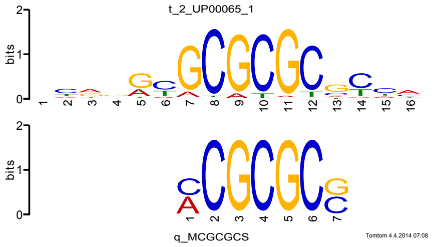

**Name**	UP00001_1
**Alt. Name**	E2F2_primary
**Database**	uniprobe_mouse.meme
**p-value**	0.00107851
**E-value**	0.929677
**q-value**	0.154898
**Overlap**	7
**Offset**	6
**Orientation**	Normal

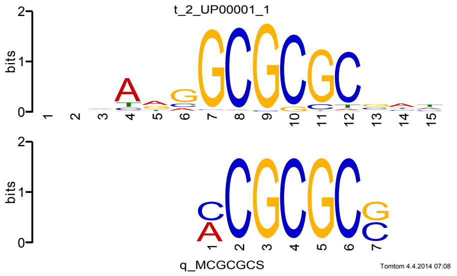

**Name**	UP00003_1
**Alt. Name**	E2F3_primary
**Database**	uniprobe_mouse.meme
**p-value**	0.00107851
**E-value**	0.929677
**q-value**	0.154898
**Overlap**	7
**Offset**	6
**Orientation**	Normal

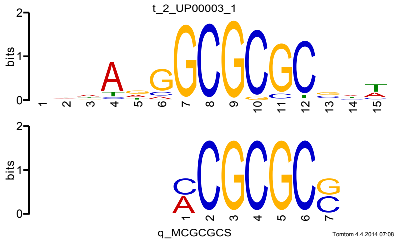

Figure S11. **TOMTOM results matching the CGCGC motif**. (A) TOMTOM top and most significant hits (RSC3/RSC30) for the CGCGC motif. The TOMTOM tool provides three significance measurements, the raw p-value, the E-value and the q-value (false discovery rate). The name of the matching protein and other summary information is presented in the left panel, while motif logos are shown on the right. The top motif logo is the target motif while the bottom is the query motif. (B) Other, less significant TOMTOM hits. Three E2f proteins are reported: E2f1/2/3. The databases that contain the CGCGC-matching motifs are Jaspar Core 2009 (E2f1) and Mouse Uniprobe (E2f2/3).

Figure S12

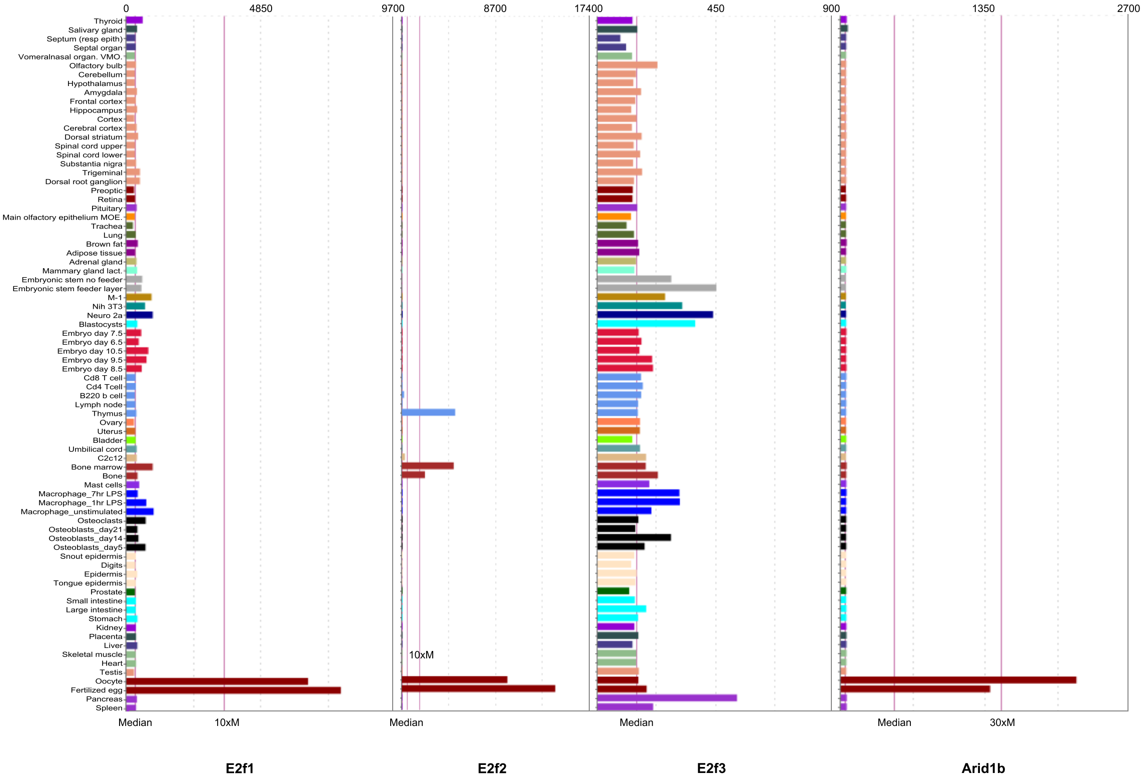

Figure S12. **Tissue-specific expression profiles for E2f1/2/3 and Arid1b**. The profiles were determined using BioGPS (http://biogps.org, [33] GeneAtlas GNF1M gcrma dataset). The x-axes represent the absolute expression level of the respective gene, normalised to be comparable across tissues. The median expression level across tissues and multiples of the median (10xM: 10x median; 30xM: 30x median) are marked by vertical purple lines. E2f1/2 and Arid1b are highly expressed specifically in oocytes, as opposed to E2f3. There was no entry for Zfp161 in the BioGPS database. The expression of these genes in oocytes also was examined using the WAMIDEX web service [34] . If the gene was in the top 20th percentile relative to other genes, then it was considered to be expressed in oocytes:

E2f1 and Arid1b are expressed in primordial, primary, secondary, small antral, large antral, germinal vesicle (GV) and metaphase II (MII) oocytes. E2f2 is expressed in primary, GV and MII oocytes. Zfp161 is expressed in GV and MII oocytes. E2f3 is not expressed at any oocyte stage.
