# Supplementary material for: Protection of CpG islands against de novo DNA methylation during oogenesis is associated with the recognition site of E2f1 and E2f2
Source: Epigenetics Chromatin. 2014 Oct 21;7:26. doi: 10.1186/1756-8935-7-26 (PMC4255709; doi:10.1186/1756-8935-7-26)
Supplement: Additional file 3 — Supplementary Results and Methods. [file 1756-8935-7-26-S3.pdf]

SUPPLEMENTARY RESULTS AND DISCUSSION

CpG spacing in permanent maternal gDMRs overall is periodic but individually highly variable

The average obs/exp ratios for distances up to 200bp for the 28 DMR CGIs form a periodic pattern with peaks at distances 9, 18, 27 and 37bp (Figure 3), in contrast to other oocyte-methylated or unmethylated CGIs. The pattern is consistent with regularly spaced CpGs ~9bp apart being enriched in DMR CGIs *relative to other distances*. This is consistent with the observed larger proportion of DMR CGIs that are significantly (empirical $p < 0.05$) enriched for pairs of CpGs at 8-10bp (37%; Figure 4.A) compared to pairs at 12-14bp (2%; Figure 4.B). In contrast, the analogous proportions for both unmethylated and other oocyte-methylated CGIs do not depend on the particular distance range (Figure 4), consistent with the lack of periodic patterns in their obs/exp rations (Figure 3).

A previous study provided evidence for the evolutionary conservation of CpGs in permanent maternal gDMRs in general, compared to permanent paternal gDMRs and typically unmethylated CGI promoters (26). Our results are consistent with this finding and in addition suggest that on average, CpG pairs 8-10bp apart are preferentially maintained in permanent maternal gDMRs.

Given the small number of permanent maternal gDMRs, we investigated them individually with respect to the spacing of their CpGs. Fourteen of the 23 permanent maternal gDMRs contain a CGI whose obs/exp ratio noticeably peaks at 8-10bp, and eight of them show a periodic pattern of peaks (Table S2). We quantified the contribution of each DMR CGI to the average obs/exp ratios and asked for each of the four peaks in the average whether or not a particular CGI made an above average contribution to the peak (Additional files 3 & 4: "Obs_exp ratios" spreadsheet). If all CGIs contributed approximately equally to all four peaks, i.e., if the average obs/exp ratios were representative, one expects a CGI to make an above average contribution to a peak 50% of the time, i.e., to two of the four peaks. However, we observed an unlikely excess of occurrences where a CGI either contributed above average to at the most one peak, or to at least three peaks (22 of 28 CGIs; binomial $P(>21)= 0.0553$ with $p=0.625$). The number of CGIs contributing above average to none or all four peaks significantly exceeded expectations (10 of 28 CGIs; binomial $P(>9)= 0.0014$ with $p=0.125$). We conclude that the average obs/exp ratios for all 28 DMR CGIs are not representative, i.e., the average is a poor predictor of periodicity for any one DMR CGI.

The obs/exp ratio for individual DMR CGIs may not exhibit a periodic pattern due to superimposition of multiple different periods. Therefore, we applied the fast Fourier transformation (FTT) to the obs/exp ratio data for each DMR CGI, with dominant periods in CpG spacing represented by high amplitude peaks in the resulting frequency spectrum. The spectrum for the average obs/exp ratios for all 28 DMR CGIs is dominated by a peak at 9.14bp with an amplitude of 0.07 (Figure S3), consistent with the periodic pattern in the obs/exp ratios of DMR CGIs (Figure 3). Individually, the spectra of CGIs in 16 permanent maternal gDMRs contain an 8-10bp period with an amplitude >0.07 (Table S2). For six of them, no other period has greater amplitude. The other permanent maternal gDMRs lack evidence of a 8-10bp period in CpG spacing by the FFT method (Table S2), consistent with results for

other methods (e.g., auto-correlation analysis: see Table S2, Figure S4.B and Supplementary Methods below for details).

In summary, the specific subset of 28 oocyte-methylated CGIs in permanent maternal gDMRs are on average relatively enriched with CpG pairs at 8-10bp (Figure 4) and on average exhibit a ~9bp period in CpG spacing (Figures 3 and S3). However, there is considerable variability between individual DMR CGIs with respect to the existence of periodic CpG spacing as well as the lengths of the present periods (Table S2; Figures S5-S7). This lack of consistency, even among CGIs belonging to the same permanent maternal gDMR, does not support the idea of periodic CpG spacing being generally involved in targeting Dnmt3a/l to these regions.

SUPPLEMENTARY METHODS

Autocorrelation coefficient (ACC) and fast Fourier transform (FFT) analysis of periodicity

Obs/exp ratios as computed in the main METHODS section were smoothed twice, each time averaging over a 3bp sliding window, to remove high frequency noise, and then de-trended to remove the approximately linear, global downward trend in the ratios.

To see whether there is a regular period in the spacing of CpGs in a CGI category, we computed auto-correlation coefficients (ACC) from the smoothed and de-trended obs/exp ratios for distances between 5 and 45bp and distances between 5+dD and 45+dD bp for dD ranging from 0 to 40bp (Figure S4.B), and for distances between 5 and 105bp and distances between 5+dD and 105+dD bp for dD ranging from 0 to 100bp (data not shown). A dominant period in the obs/exp ratio data with a wave length of, for example, 9bp results in the ACC peaking at dD= 9bp and multiples of 9bp. The results using the ACC method were not qualitatively different from those obtained by direct observation of the obs/exp ratios (Figure S4.A, and Table S2).

The obs/exp and ACC approaches to analysing periodicity are both limited to the detection of only a single dominant period. To overcome this limitation, we applied the fast Fourier transformation (FFT) to the smoothed and de-trended obs/exp ratios for distances from 5 to 68bp for each DMR CGI and, analogously, to the smoothed and de-trended average obs/exp ratios for all DMR CGIs. The fast Fourier transform is used to decompose complex signals formed of multiple periodic signals into its constituent parts, highlighting the dominant, most powerful frequencies.

SUPPLEMENTARY REFERENCES

26. Schulz R, Proudhon C, Bestor TH, Woodfine K, Lin C-S, Lin S-P, Prissette M, Oakey RJ, Bourc'his D: **The parental non-equivalence of imprinting control regions during mammalian development and evolution.** *PLoS Genet* 2010, **6:**e1001214.
