# Supplementary material for: Protection of CpG islands against de novo DNA methylation during oogenesis is associated with the recognition site of E2f1 and E2f2
Source: Epigenetics Chromatin. 2014 Oct 21;7:26. doi: 10.1186/1756-8935-7-26 (PMC4255709; doi:10.1186/1756-8935-7-26)
Supplement: Additional file 4 — Description of supplementary spreadsheets. [file 1756-8935-7-26-S4.pdf]

CGIs_transcripts_Annotation sheet: 28,781 CGI-transcript pairs

Column Header	Description
G_ID	CGI identification number from Smallwood et al., (2011)
CGI_Chr	Chromosome
CGI_Start	Start
CGI_End	End
Anno_Name	Symbol of CGI-associated gene
Anno_Promoter	Number of annotated promoters that overlap the CGI (Smallwood et al., 2011)
H3K4me3_LogFC<br>H3K4me3_Pvalue<br>H3K4me3_Adj_Pvalue	H3k4me3 ChIP-seq signal statistics for the CGI
Cuff_trans_id	Cufflinks-assigned transcript id
Ref_trans_id	Cufflinks-assigned reference transcript id
Transcript_Chr	Chromosome
Transcript_Start	Start
Transcript_End	End
Strand	Strand
Class_code	Cufflinks transcript classification (novel, overlap, etc.)
FPKM	Cufflinks Fragments Per Kilobase of transcript per Million mapped reads
Class	Our CGI classification
Anno_Class	CGI classification relative to annotated genes (Smallwood et al., 2011; Annotated_Promoter "sixth column"):<br>0: Intragenic<br>>0: Promoter-associated<br>Null: Intergenic
DMR CGIs	Overlap with permanent maternal gDMRs (Yes/No)
Goocyte_Methy	Oocyte-methylated CGI according to Smallwood et al., 2011 and consistent with Kobayashi et al., 2012 (Yes/No)
Goocyte_Unmethy	Oocyte-un-methylated CGI according to Smallwood et al., 2011 and consistent with Kobayashi et al., 2012 (Yes/No)
GK_Blast	Methylated CGI in Blastocyst according to Smallwood et al., 2011 and consistent with Kobayashi et al., 2012 (Yes/No)
K_ESCs	Methylated CGI in ESCs according to Kobayashi et al., 2012 (Yes/No)
MeDIP500_FDR<br>MeDIP500_Log2	Overlap with significantly (FDR< 50%) MeDIP-enriched genomic region using 500bp sliding window size; FDR and log2 fold change of region (Proudhon et al., 2012)
MedIP1000_FDR<br>MedIP1000_Log2	Overlap with significantly (FDR< 50%) MeDIP-enriched genomic region using 1000bp sliding window size; FDR and log2 fold change of region (Proudhon et al., 2012)
E85_WT_Abs	Absolute log methylation levels of CGI in wild type E8.5 mouse embryo (Proudhon et al., 2012)
E85_MUT_Abs	Absolute log methylation levels of CGI in mutant E8.5 mouse embryo (Proudhon et al., 2012)
E85_Relative_FDR<br>E85_Relative_Log2	FDR and log2 fold change for CGI in mutant relative to WT (Proudhon et al., 2012)
Adult_Liver	Absolute log methylation levels of CGI in mouse liver (Proudhon et al., 2012)
LogFC_TSS<br>Pvalue_TSS<br>Adj_Pvalue_TSS	H3k4me3 ChIP-seq signal statistics for the CGI-associated promoter

CGIs_methylation_Annotation sheet: 23,020 CGIs

Column Header	Description
**G_ID**	CGI identification number from Smallwood et al., (2011)
**B_ID**	CGI identification number from Illingworth et al., (2010)
**Chr**	Coordinates based on Smallwood et al., (2011) annotation
**Start**	
**End**	
**Name**	Symbol of CGI-associated gene
**DMR CGIs**	Overlap with permanent maternal gDMRs (Yes/No)
**Goocyte_Methy**	Oocyte-methylated CGI according to Smallwood et al., 2011 and consistent with Kobayashi et al., 2012 (Yes/No)
**Goocyte_Unmethy**	Oocyte-un-methylated CGI according to Smallwood et al., 2011 and consistent with Kobayashi et al., 2012 (Yes/No)
**GK_Blast**	Methylated CGI in Blastocyst according to Smallwood et al., 2011 and consistent with Kobayashi et al., 2012 (Yes/No)
**K_ESCs**	Methylated CGI in ESCs according to Kobayashi et al., 2012 (Yes/No)
**MeDIP500_FDR**	Overlap with significantly (FDR< 50%) MeDIP-enriched genomic region using 500bp sliding window size; FDR and log2 fold change of region (Proudhon et al., 2012)
**MeDIP500_Log2**	
**MedIP1000_FDR**	Overlap with significantly (FDR< 50%) MeDIP-enriched genomic region using 1000bp sliding window size; FDR and log2 fold change of region (Proudhon et al., 2012)
**MedIP1000_Log2**	
**E85_WT_Abs**	Absolute log methylation levels of CGI in wild type E8.5 mouse embryo (Proudhon et al., 2012)
**E85_MUT_Abs**	Absolute log methylation levels of CGI in mutant E8.5 mouse embryo (Proudhon et al., 2012)
**E85_Relative_FDR**	FDR and log2 fold change for CGI in mutant relative to WT (Proudhon et al., 2012)
**E85_Relative_Log2**	
**Adult_Liver**	Absolute log methylation levels of CGI in mouse liver (Proudhon et al., 2012)
**Zfp57_ID**	Zfp57 ChIP-seq id from Quenneville et al. 2011
**Min_Distance**	Minimum distance from the nearest Zfp57 ChIP-seq peak; Quenneville et al. 2011
**TGCCGC**	Number of TGCCGC occurrences within the CGI
**TGCCGC_1kb**	Number of TGCCGC occurrences within the CGI +/- 1kb flanking sequences

Un-methylated associated Factor sheet: 23020 CGIs

**ID**	Sequential identification number
**G_ID**	CGI identification number from Smallwood et al., (2011)
**PA**	Promoter-associated during early oocyte growth (Yes: 1 / No: 0)
**Methy_State**	CGI methylation state (Methy / Unmethy / NULL)
**Motif**	Contains CGCGC motif (Yes: 1 / No: 0)
**Cfp1**	Overlaps Cfp1 peak from Thomson et al., (2010) (Yes: 1 / No: 0)
**Rloop**	R-loop potential (Yes: 1 / Unknown: 0 / No: -1)
**H3K4me3**	Overlaps H3K4me3 peak from Smallwood et al., (2011) (Yes: 1 / No: 0)

Obs_exp ratios sheet: observed / expected ratios of CpG pairs for DMR CGIs for distances between CpGs up to 40 bp
