# Supplementary material for: Protection of CpG islands against de novo DNA methylation during oogenesis is associated with the recognition site of E2f1 and E2f2
Source: Epigenetics Chromatin. 2014 Oct 21;7:26. doi: 10.1186/1756-8935-7-26 (PMC4255709; doi:10.1186/1756-8935-7-26)
Supplement: Additional file 6 — Mini-website with GEM peak calling and motif analysis results for E2F1 ChIP-seq in MCF7 cells. [file 1756-8935-7-26-S6.zip › E2F1_MCF7_GEM_outputs/E2F1_shuffle_121_hg19_3RDistro_1_result.html]

E2F1\_shuffle\_121\_hg19\_3RDistro\_1 | || Significant Events  : 28944 Insignificant Events: 22163 Filtered Events      : 205 Total positive sequences: 5000 - Complete KSM (K-mer Set Motif) file.- K-mer alignment file.- Motif PFMs   K-mer | Cluster | Offset | Pos Hit | Neg Hit | HGP || **-GGCGCGC** | 0 | -3 | 630 | 171 | -67.7 | | **--GCGCGCT** | 0 | -2 | 445 | 101 | -55.5 | | **--GCGCGCA** | 0 | -2 | 394 | 93 | -47.4 | | **-GGCGCTC** | 0 | -3 | 360 | 100 | -37.2 | | **--GCGCTCT** | 0 | -2 | 269 | 60 | -34.0 | | **-GACGCGC** | 0 | -3 | 197 | 64 | -17.4 | | **GGCGCCA** | 1 | -4 | 453 | 92 | -61.6 | | **-GCGCCAG** | 1 | -3 | 461 | 114 | -53.4 | | **GGCGCCT** | 1 | -4 | 496 | 148 | -47.7 | | **-GCGCCTT** | 1 | -3 | 278 | 61 | -35.5 | | **-GCGCCAA** | 1 | -3 | 225 | 38 | -34.6 | | **AGCGCCA** | 1 | -4 | 263 | 71 | -28.1 | | **-GCGCCTA** | 1 | -3 | 117 | 37 | -11.1 | | **-GCGGGAA** | 2 | -3 | 402 | 111 | -41.8 | | **-GCGCGAA** | 2 | -3 | 126 | 37 | -12.9 | | **-----GCGCCCT** | 3 | 1 | 389 | 116 | -37.3 | | **-----GCGCACT** | 3 | 1 | 141 | 38 | -15.5 | | Motif PWM | Motif spatial distribution (w.r.t. primary PWM) Format: position,motif\_occurences || rc PWM: 7.35/11.70, hit=1332+/506-, hgp=1e-103.7 |  | | rc PWM: 7.10/11.76, hit=1453+/626-, hgp=1e-94.2 |  | | rc PWM: 8.40/13.22, hit=436+/149-, hgp=1e-35.6 |  | | rc PWM: 7.54/12.54, hit=381+/147-, hgp=1e-26.2 |  | |
