# Supplementary material for: Protection of CpG islands against de novo DNA methylation during oogenesis is associated with the recognition site of E2f1 and E2f2
Source: Epigenetics Chromatin. 2014 Oct 21;7:26. doi: 10.1186/1756-8935-7-26 (PMC4255709; doi:10.1186/1756-8935-7-26)
Supplement: Additional file 6 — Mini-website with GEM peak calling and motif analysis results for E2F1 ChIP-seq in MCF7 cells. [file 1756-8935-7-26-S6.zip › E2F1_MCF7_GEM_outputs/index.html]

E2F1\_shuffle\_121\_hg19\_3RDistro\_2 | || Significant Events  : 30467 Insignificant Events: 17549 Filtered Events      : 31 Total positive sequences: 5000 - Complete KSM (K-mer Set Motif) file.- K-mer alignment file.- Motif PFMs   K-mer | Cluster | Offset | Pos Hit | Neg Hit | HGP || **-GGCGCGC** | 0 | -4 | 880 | 171 | -128.0 | | **--GCGCGCT** | 0 | -3 | 605 | 101 | -94.7 | | **--GCGCGCA** | 0 | -3 | 530 | 93 | -80.0 | | **-GGCGCTC** | 0 | -4 | 522 | 100 | -74.3 | | **---CGCGCGG** | 0 | -2 | 586 | 158 | -63.1 | | **--GCGCTCT** | 0 | -3 | 378 | 60 | -60.2 | | **CGGCGCG** | 0 | -5 | 604 | 179 | -59.0 | | **--GCGCTCG** | 0 | -3 | 329 | 84 | -36.9 | | **--GCGCCAG** | 1 | -3 | 502 | 114 | -62.7 | | **-GGCGCCA** | 1 | -4 | 426 | 92 | -55.2 | | **-CGCGCCT** | 1 | -4 | 452 | 130 | -45.2 | | **-GGCGCCT** | 1 | -4 | 461 | 148 | -40.7 | | **-GCGGCGC** | 2 | -4 | 706 | 213 | -68.3 | | **--CGGCGCG** | 2 | -3 | 604 | 179 | -59.0 | | **--GCGGGAA** | 3 | -3 | 409 | 111 | -43.3 | | **-GCGCGCA** | 4 | -4 | 530 | 93 | -80.0 | | **--CGCGCAG** | 4 | -3 | 446 | 122 | -46.9 | | **--CGCGCAC** | 4 | -3 | 319 | 81 | -36.0 | | **----GCGCCCT** | 5 | -1 | 420 | 116 | -43.7 | | **--CCTGGCG** | 7 | -3 | 419 | 119 | -42.4 | | Motif PWM | Motif spatial distribution (w.r.t. primary PWM) Format: position,motif\_occurences || rc PWM: 7.19/11.71, hit=1990+/506-, hgp=1e-271.3 |  | | rc PWM: 6.42/10.41, hit=1495+/660-, hgp=1e-93.4 |  | | rc PWM: 7.19/11.95, hit=807+/375-, hgp=1e-41.4 |  | | rc PWM: 8.39/13.22, hit=453+/151-, hgp=1e-38.2 |  | | rc PWM: 7.58/12.59, hit=414+/155-, hgp=1e-29.8 |  | | rc PWM: 8.96/12.98, hit=277+/96-, hgp=1e-22.3 |  | | rc PWM: 8.03/13.27, hit=308+/121-, hgp=1e-20.7 |  | | rc PWM: 9.39/12.97, hit=254+/90-, hgp=1e-19.9 |  | |
