# Supplementary figures and images for: Protection of CpG islands against de novo DNA methylation during oogenesis is associated with the recognition site of E2f1 and E2f2
Source: Epigenetics Chromatin. 2014 Oct 21;7:26. doi: 10.1186/1756-8935-7-26 (PMC4255709; doi:10.1186/1756-8935-7-26)

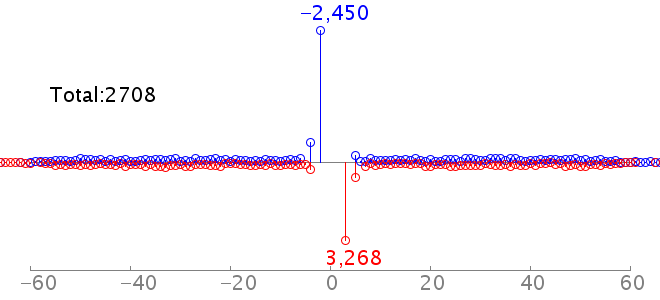

Supplement: Additional file 6 — Mini-website with GEM peak calling and motif analysis results for E2F1 ChIP-seq in MCF7 cells. [file 1756-8935-7-26-S6.zip › E2F1_MCF7_GEM_outputs/E2F1_shuffle_121_hg19_3RDistro_2_Spatial_dist_0_2.png]

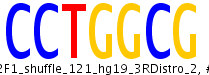

Supplement: Additional file 6 — Mini-website with GEM peak calling and motif analysis results for E2F1 ChIP-seq in MCF7 cells. [file 1756-8935-7-26-S6.zip › E2F1_MCF7_GEM_outputs/E2F1_shuffle_121_hg19_3RDistro_2_7_motif.png]

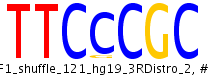

Supplement: Additional file 6 — Mini-website with GEM peak calling and motif analysis results for E2F1 ChIP-seq in MCF7 cells. [file 1756-8935-7-26-S6.zip › E2F1_MCF7_GEM_outputs/E2F1_shuffle_121_hg19_3RDistro_2_3_motif_rc.png]

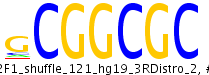

Supplement: Additional file 6 — Mini-website with GEM peak calling and motif analysis results for E2F1 ChIP-seq in MCF7 cells. [file 1756-8935-7-26-S6.zip › E2F1_MCF7_GEM_outputs/E2F1_shuffle_121_hg19_3RDistro_2_2_motif.png]

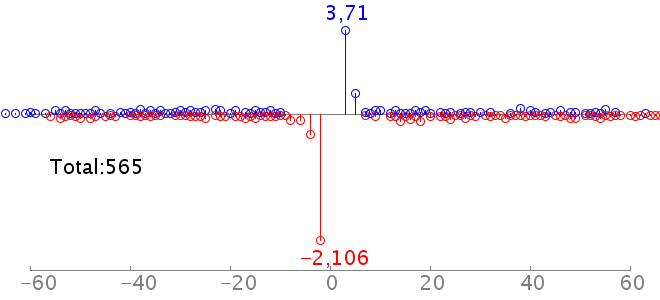

Supplement: Additional file 6 — Mini-website with GEM peak calling and motif analysis results for E2F1 ChIP-seq in MCF7 cells. [file 1756-8935-7-26-S6.zip › E2F1_MCF7_GEM_outputs/E2F1_shuffle_121_hg19_3RDistro_2_Spatial_dist_0_5.png]

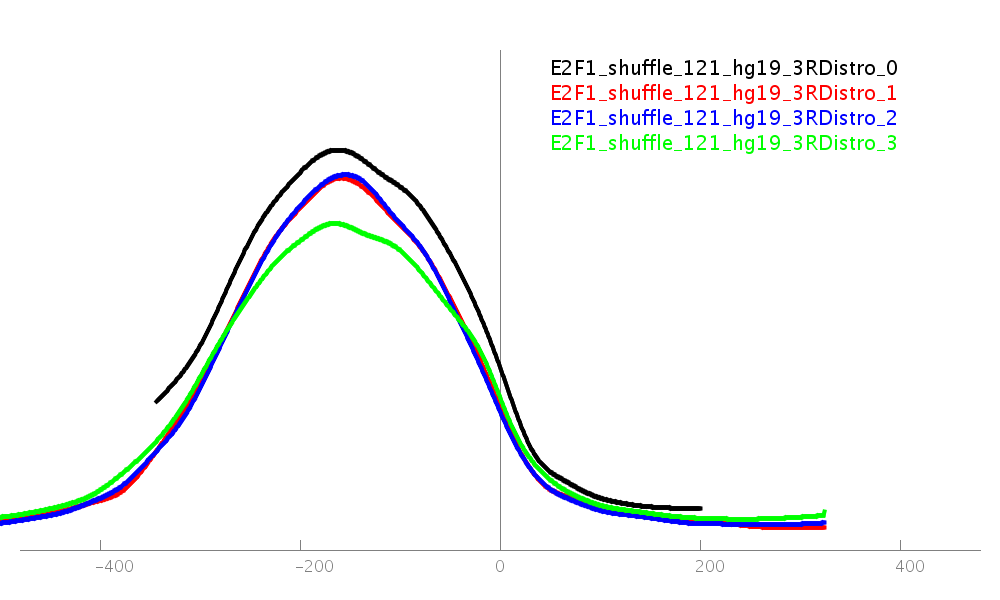

Supplement: Additional file 6 — Mini-website with GEM peak calling and motif analysis results for E2F1 ChIP-seq in MCF7 cells. [file 1756-8935-7-26-S6.zip › E2F1_MCF7_GEM_outputs/E2F1_shuffle_121_hg19_3RDistro_All_Read_Distributions.png]

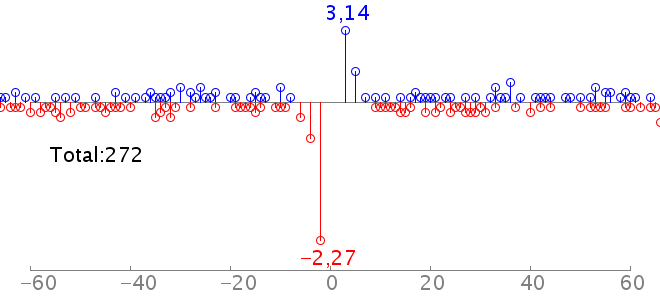

Supplement: Additional file 6 — Mini-website with GEM peak calling and motif analysis results for E2F1 ChIP-seq in MCF7 cells. [file 1756-8935-7-26-S6.zip › E2F1_MCF7_GEM_outputs/E2F1_shuffle_121_hg19_3RDistro_1_Spatial_dist_0_2.png]

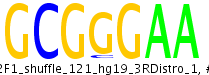

Supplement: Additional file 6 — Mini-website with GEM peak calling and motif analysis results for E2F1 ChIP-seq in MCF7 cells. [file 1756-8935-7-26-S6.zip › E2F1_MCF7_GEM_outputs/E2F1_shuffle_121_hg19_3RDistro_1_2_motif.png]

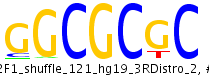

Supplement: Additional file 6 — Mini-website with GEM peak calling and motif analysis results for E2F1 ChIP-seq in MCF7 cells. [file 1756-8935-7-26-S6.zip › E2F1_MCF7_GEM_outputs/E2F1_shuffle_121_hg19_3RDistro_2_0_motif.png]

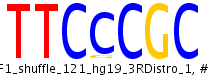

Supplement: Additional file 6 — Mini-website with GEM peak calling and motif analysis results for E2F1 ChIP-seq in MCF7 cells. [file 1756-8935-7-26-S6.zip › E2F1_MCF7_GEM_outputs/E2F1_shuffle_121_hg19_3RDistro_1_2_motif_rc.png]

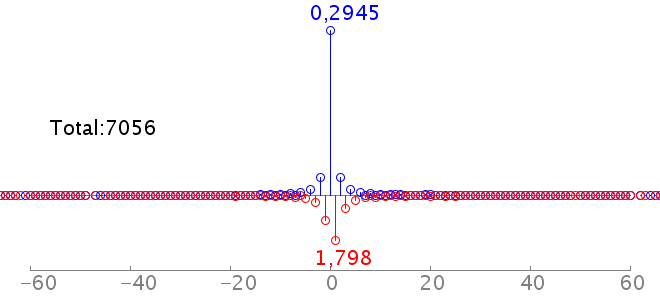

Supplement: Additional file 6 — Mini-website with GEM peak calling and motif analysis results for E2F1 ChIP-seq in MCF7 cells. [file 1756-8935-7-26-S6.zip › E2F1_MCF7_GEM_outputs/E2F1_shuffle_121_hg19_3RDistro_1_Spatial_dist_0_0.png]

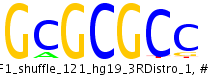

Supplement: Additional file 6 — Mini-website with GEM peak calling and motif analysis results for E2F1 ChIP-seq in MCF7 cells. [file 1756-8935-7-26-S6.zip › E2F1_MCF7_GEM_outputs/E2F1_shuffle_121_hg19_3RDistro_1_0_motif_rc.png]

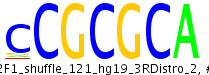

Supplement: Additional file 6 — Mini-website with GEM peak calling and motif analysis results for E2F1 ChIP-seq in MCF7 cells. [file 1756-8935-7-26-S6.zip › E2F1_MCF7_GEM_outputs/E2F1_shuffle_121_hg19_3RDistro_2_4_motif.png]

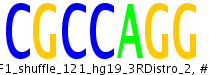

Supplement: Additional file 6 — Mini-website with GEM peak calling and motif analysis results for E2F1 ChIP-seq in MCF7 cells. [file 1756-8935-7-26-S6.zip › E2F1_MCF7_GEM_outputs/E2F1_shuffle_121_hg19_3RDistro_2_7_motif_rc.png]

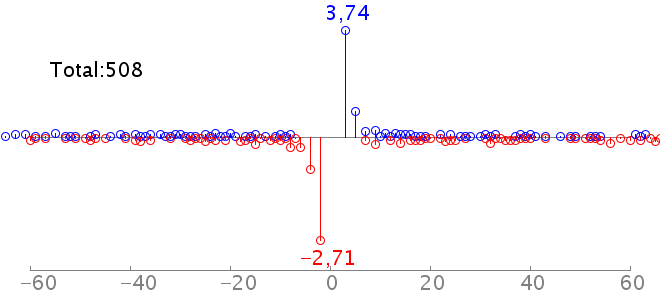

Supplement: Additional file 6 — Mini-website with GEM peak calling and motif analysis results for E2F1 ChIP-seq in MCF7 cells. [file 1756-8935-7-26-S6.zip › E2F1_MCF7_GEM_outputs/E2F1_shuffle_121_hg19_3RDistro_1_Spatial_dist_0_3.png]

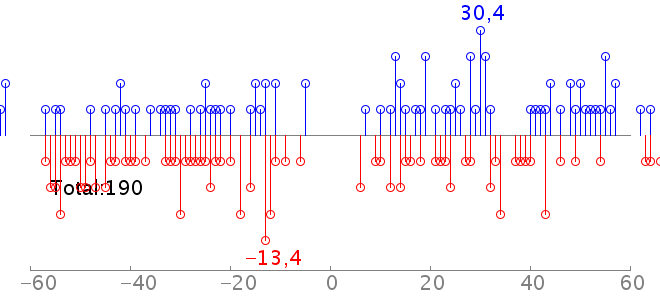

Supplement: Additional file 6 — Mini-website with GEM peak calling and motif analysis results for E2F1 ChIP-seq in MCF7 cells. [file 1756-8935-7-26-S6.zip › E2F1_MCF7_GEM_outputs/E2F1_shuffle_121_hg19_3RDistro_2_Spatial_dist_0_6.png]

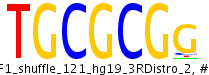

Supplement: Additional file 6 — Mini-website with GEM peak calling and motif analysis results for E2F1 ChIP-seq in MCF7 cells. [file 1756-8935-7-26-S6.zip › E2F1_MCF7_GEM_outputs/E2F1_shuffle_121_hg19_3RDistro_2_4_motif_rc.png]

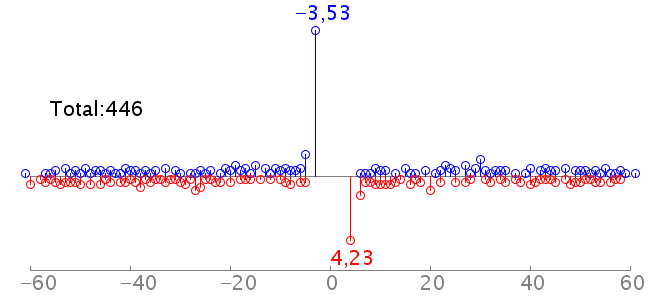

Supplement: Additional file 6 — Mini-website with GEM peak calling and motif analysis results for E2F1 ChIP-seq in MCF7 cells. [file 1756-8935-7-26-S6.zip › E2F1_MCF7_GEM_outputs/E2F1_shuffle_121_hg19_3RDistro_2_Spatial_dist_0_7.png]

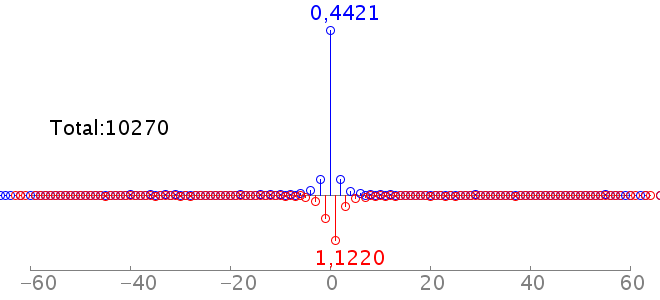

Supplement: Additional file 6 — Mini-website with GEM peak calling and motif analysis results for E2F1 ChIP-seq in MCF7 cells. [file 1756-8935-7-26-S6.zip › E2F1_MCF7_GEM_outputs/E2F1_shuffle_121_hg19_3RDistro_2_Spatial_dist_0_0.png]

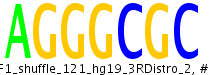

Supplement: Additional file 6 — Mini-website with GEM peak calling and motif analysis results for E2F1 ChIP-seq in MCF7 cells. [file 1756-8935-7-26-S6.zip › E2F1_MCF7_GEM_outputs/E2F1_shuffle_121_hg19_3RDistro_2_5_motif_rc.png]

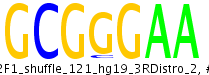

Supplement: Additional file 6 — Mini-website with GEM peak calling and motif analysis results for E2F1 ChIP-seq in MCF7 cells. [file 1756-8935-7-26-S6.zip › E2F1_MCF7_GEM_outputs/E2F1_shuffle_121_hg19_3RDistro_2_3_motif.png]

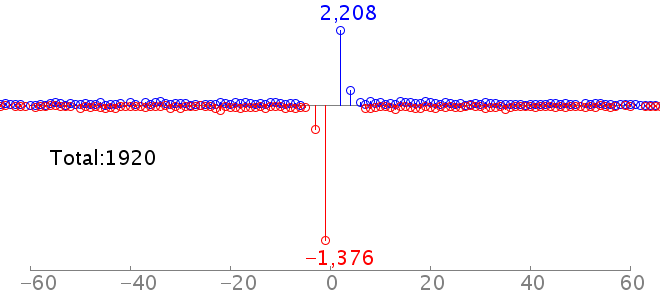

Supplement: Additional file 6 — Mini-website with GEM peak calling and motif analysis results for E2F1 ChIP-seq in MCF7 cells. [file 1756-8935-7-26-S6.zip › E2F1_MCF7_GEM_outputs/E2F1_shuffle_121_hg19_3RDistro_1_Spatial_dist_0_1.png]

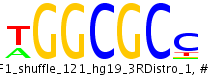

Supplement: Additional file 6 — Mini-website with GEM peak calling and motif analysis results for E2F1 ChIP-seq in MCF7 cells. [file 1756-8935-7-26-S6.zip › E2F1_MCF7_GEM_outputs/E2F1_shuffle_121_hg19_3RDistro_1_1_motif_rc.png]

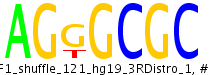

Supplement: Additional file 6 — Mini-website with GEM peak calling and motif analysis results for E2F1 ChIP-seq in MCF7 cells. [file 1756-8935-7-26-S6.zip › E2F1_MCF7_GEM_outputs/E2F1_shuffle_121_hg19_3RDistro_1_3_motif_rc.png]

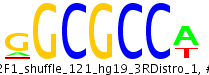

Supplement: Additional file 6 — Mini-website with GEM peak calling and motif analysis results for E2F1 ChIP-seq in MCF7 cells. [file 1756-8935-7-26-S6.zip › E2F1_MCF7_GEM_outputs/E2F1_shuffle_121_hg19_3RDistro_1_1_motif.png]

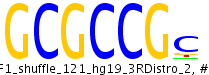

Supplement: Additional file 6 — Mini-website with GEM peak calling and motif analysis results for E2F1 ChIP-seq in MCF7 cells. [file 1756-8935-7-26-S6.zip › E2F1_MCF7_GEM_outputs/E2F1_shuffle_121_hg19_3RDistro_2_2_motif_rc.png]

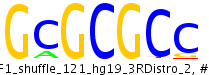

Supplement: Additional file 6 — Mini-website with GEM peak calling and motif analysis results for E2F1 ChIP-seq in MCF7 cells. [file 1756-8935-7-26-S6.zip › E2F1_MCF7_GEM_outputs/E2F1_shuffle_121_hg19_3RDistro_2_0_motif_rc.png]

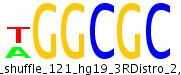

Supplement: Additional file 6 — Mini-website with GEM peak calling and motif analysis results for E2F1 ChIP-seq in MCF7 cells. [file 1756-8935-7-26-S6.zip › E2F1_MCF7_GEM_outputs/E2F1_shuffle_121_hg19_3RDistro_2_1_motif_rc.png]

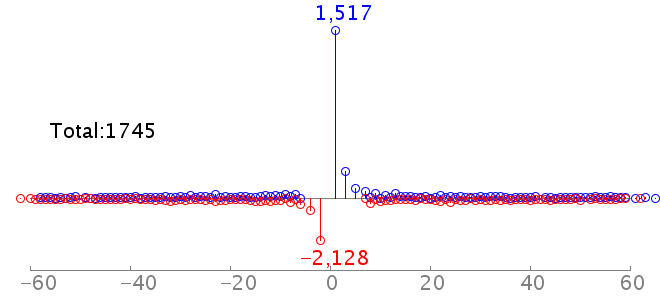

Supplement: Additional file 6 — Mini-website with GEM peak calling and motif analysis results for E2F1 ChIP-seq in MCF7 cells. [file 1756-8935-7-26-S6.zip › E2F1_MCF7_GEM_outputs/E2F1_shuffle_121_hg19_3RDistro_2_Spatial_dist_0_4.png]

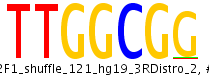

Supplement: Additional file 6 — Mini-website with GEM peak calling and motif analysis results for E2F1 ChIP-seq in MCF7 cells. [file 1756-8935-7-26-S6.zip › E2F1_MCF7_GEM_outputs/E2F1_shuffle_121_hg19_3RDistro_2_6_motif.png]

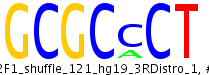

Supplement: Additional file 6 — Mini-website with GEM peak calling and motif analysis results for E2F1 ChIP-seq in MCF7 cells. [file 1756-8935-7-26-S6.zip › E2F1_MCF7_GEM_outputs/E2F1_shuffle_121_hg19_3RDistro_1_3_motif.png]

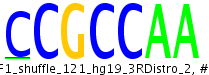

Supplement: Additional file 6 — Mini-website with GEM peak calling and motif analysis results for E2F1 ChIP-seq in MCF7 cells. [file 1756-8935-7-26-S6.zip › E2F1_MCF7_GEM_outputs/E2F1_shuffle_121_hg19_3RDistro_2_6_motif_rc.png]

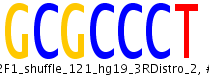

Supplement: Additional file 6 — Mini-website with GEM peak calling and motif analysis results for E2F1 ChIP-seq in MCF7 cells. [file 1756-8935-7-26-S6.zip › E2F1_MCF7_GEM_outputs/E2F1_shuffle_121_hg19_3RDistro_2_5_motif.png]

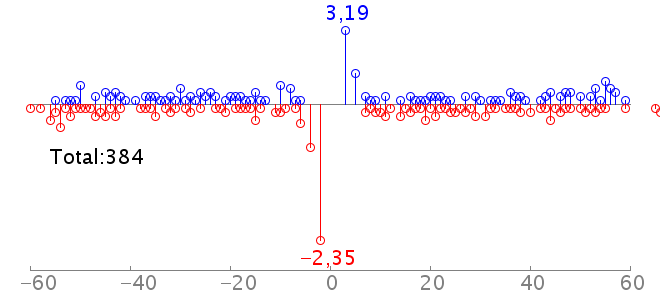

Supplement: Additional file 6 — Mini-website with GEM peak calling and motif analysis results for E2F1 ChIP-seq in MCF7 cells. [file 1756-8935-7-26-S6.zip › E2F1_MCF7_GEM_outputs/E2F1_shuffle_121_hg19_3RDistro_2_Spatial_dist_0_3.png]

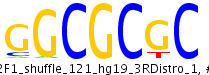

Supplement: Additional file 6 — Mini-website with GEM peak calling and motif analysis results for E2F1 ChIP-seq in MCF7 cells. [file 1756-8935-7-26-S6.zip › E2F1_MCF7_GEM_outputs/E2F1_shuffle_121_hg19_3RDistro_1_0_motif.png]

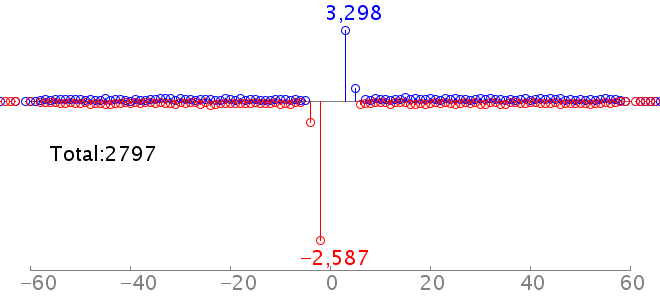

Supplement: Additional file 6 — Mini-website with GEM peak calling and motif analysis results for E2F1 ChIP-seq in MCF7 cells. [file 1756-8935-7-26-S6.zip › E2F1_MCF7_GEM_outputs/E2F1_shuffle_121_hg19_3RDistro_2_Spatial_dist_0_1.png]

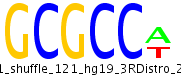

Supplement: Additional file 6 — Mini-website with GEM peak calling and motif analysis results for E2F1 ChIP-seq in MCF7 cells. [file 1756-8935-7-26-S6.zip › E2F1_MCF7_GEM_outputs/E2F1_shuffle_121_hg19_3RDistro_2_1_motif.png]
